# Sensational placodes: Neurogenesis in the otic and olfactory systems

**DOI:** 10.1016/j.ydbio.2014.01.023

**Published:** 2014-05-01

**Authors:** Esther C. Maier, Ankur Saxena, Berta Alsina, Marianne E. Bronner, Tanya T. Whitfield

**Affiliations:** a MRC Centre for Developmental and Biomedical Genetics and Department of Biomedical Science, University of Sheffield, Sheffield, S10 2TN, UK; b Division of Biology and Biological Engineering, MC 139-74, California Institute of Technology, Pasadena, CA 91125, USA; c Laboratory of Developmental Biology, Universitat Pompeu Fabra/PRBB, Dr. Aiguader 88, 08003 Barcelona, Spain

**Keywords:** Otic, Olfactory, Neurogenesis, Hair cell, Sensory neuron

## Abstract

For both the intricate morphogenetic layout of the sensory cells in the ear and the elegantly radial arrangement of the sensory neurons in the nose, numerous signaling molecules and genetic determinants are required in concert to generate these specialized neuronal populations that help connect us to our environment. In this review, we outline many of the proteins and pathways that play essential roles in the differentiation of otic and olfactory neurons and their integration into their non-neuronal support structures. In both cases, well-known signaling pathways together with region-specific factors transform thickened ectodermal placodes into complex sense organs containing numerous, diverse neuronal subtypes. Olfactory and otic placodes, in combination with migratory neural crest stem cells, generate highly specialized subtypes of neuronal cells that sense sound, position and movement in space, odors and pheromones throughout our lives.

## Highlights

• Otic and olfactory placodes generate complex organs with a variety of cell types.
• An anterior neurogenic domain is established early in otic and olfactory placodes.
• Neural crest cells contribute primarily to glial cells in the ear and nose.
• Otic and olfactory neurogenesis is regulated by extrinsic and intrinsic factors.
• Sensory cells in the ear and nose differ in their regenerative potential.

## Introduction

**Fig. 1:**
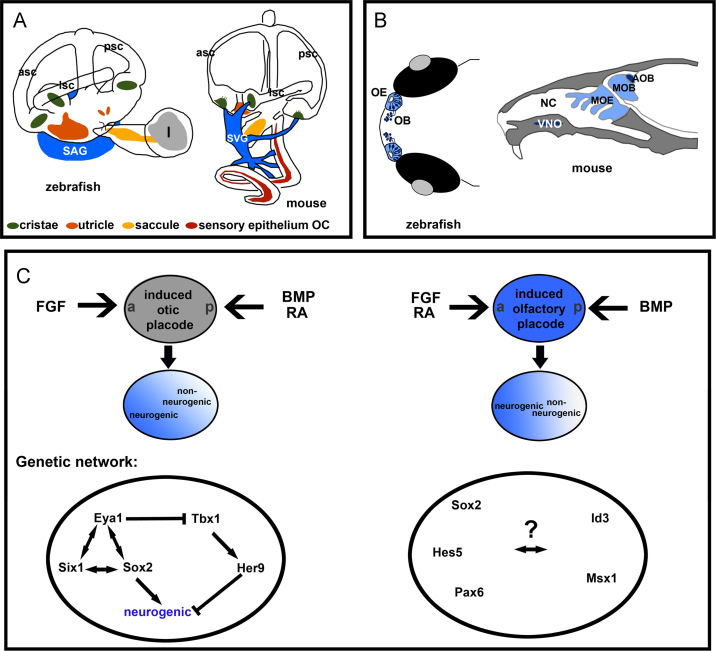
(A) Schematic drawing of an adult zebrafish and mouse inner ear, their sensory patches and approximate position of the VIIIth ganglion (SAG). (B) Schematic drawing of the olfactory system in an adult zebrafish (dorsal view) and mouse (lateral view; sagittal section). (C) Signals and genetic networks involved in the establishment of the neurogenic region in the otic (left) and olfactory (right) placode during development. Abbreviations: a: anterior, AOB: accessory olfactory bulb, asc: anterior semicircular canal, l: lagena, lsc: lateral semicircular canal, MOE: main olfactory epithelium, MOB: main olfactory bulb, NC: nasal cavity, OB: olfactory bulb, OC: organ of Corti in the cochlea, OE: olfactory epithelium, p: posterior, psc: posterior semicircular canal, SAG: statoacoustic ganglion (VIIIth ganglion), SVG: spiral and vestibular ganglia (VIIIth ganglion), and VNO: vomeronasal organ.

The vertebrate ear and nose share interesting similarities as well as important differences in their modes of sensation. Both sensory systems can detect a vast array of distinct environmental stimuli: the human nose can detect up to 400,000 different odors (Mori et al., 2006), while the inner ear can distinguish a wide range of sounds by amplitude, quality and frequency, and detect different vestibular stimuli (gravity, and linear and angular acceleration). However, the strategies employed by the nose and the inner ear differ significantly regarding the modes of transmission to the brain and discrimination between distinct inputs. Whereas olfactory sensory neurons (OSNs) are primary sensory receptor cells, with axons that project directly to the olfactory bulb, hair cells of the inner ear are secondary sensory receptor cells, lacking an axon. Hair cells convey sound and balance information to the brain indirectly via afferent auditory and vestibular neurons in the VIIIth ganglion (ganglion of the VIIIth (statoacoustic) cranial nerve) (Fig. 1A and B). For the purposes of this review, we will consider both otic neurons and sensory hair cells as neuronal cell types, and use the terms neurogenesis and sensorigenesis, respectively, to describe their generation. Otic and olfactory neurogenesis are highly similar processes, each following a sequence of commitment to a neural fate, initiation of neurogenic divisions to generate neuronal precursors and differentiation via the expression of bHLH genes.

In the olfactory system, sensory neurons are specialized to each detect a single odorant. In the mouse, for example, there are 1200 distinct odorant genes, with generally only one (or a few) expressed by each OSN, thus determining the cell׳s spectral response (Buck and Axel, 1991). No such receptor specificity exists in the inner ear hair cells. Instead, mechanical forces displace specialized stereociliary bundles such that deflection of the bundles opens ion channels that modulate the cells׳ membrane potential (Corey, 2009). In the ear, sensory epithelia responsible for detection of auditory and vestibular inputs are segregated into distinct compartments, and along the mammalian cochlea, differences in frequency sensitivity generate a tonotopic map that in turn is transmitted to the brain. Here, we review current knowledge of the process of development of the olfactory and otic systems, describing their common traits and highlighting their interesting differences, particularly with respect to neurogenesis. We also refer the reader to a number of previous excellent reviews (Fritzsch et al., 2006a, 2006b, 2010; Kelley, 2006b, 2006a, 2009; Sanchez-Calderon et al., 2007; Alsina et al., 2009; DeMaria and Ngai, 2010; Gokoffski et al., 2010; Treloar et al., 2010; Bazáes et al., 2013; Miyasaka et al., 2013; Rodriguez, 2013).

## Origin of olfactory and otic placodes

In vertebrates, all peripheral sensory neurons arise from two specialized regions of ectoderm that form at the border of the neural plate: the cranial placodes and the neural crest. Placodes are regions of condensed ectoderm arising in the head region that give rise to both non-neural (lens and adenohypophyseal placodes) and neural structures. The neurogenic placodes include the olfactory, trigeminal, profundal, lateral line, otic and epibranchial placodes (Schlosser, 2010). Most neurogenic placodes give rise solely to sensory neurons and associated structures. By contrast, the olfactory and otic placodes give rise to both neural and non-neural structures in the nose and the inner ear, respectively. Moreover, throughout development, both organs undergo a series of cellular rearrangements that spatially distribute their emerging cell types in precise anatomical positions.

Development of the placode-derived sensory organs is a multi-step process. It starts with subdivision of the embryonic ectoderm into epidermal ectoderm, neural ectoderm and the neural plate border region, as reviewed in Groves and LaBonne (this issue). While anterior placodal cells (e.g. olfactory, adenohypophyseal, and lens) express *Six3*, *Pax6* and *Otx2* and emerge in the late gastrula (Ahrens and Schlosser, 2005; Sjödal et al., 2007), posterior placodal cells (e.g. otic and epibranchial) express *Irx3*, *Pax2* and *Gbx2*, and emerge later at the neurula stage in the lateral posterior neural border (reviewed in Schlosser (2006)). Thus, induction of olfactory and otic placodes occurs at different time points and locations in the early embryo. After induction, both olfactory and otic placodes invaginate (or cavitate in zebrafish), transforming from a thickened sheet of ectoderm into a pit and then an epithelial vesicle that is initially a single cell layer thick. Subsequently, the olfactory and otic epithelia transform into multilayered structures and undergo neurogenesis. The otic placode must undergo further extensive morphogenesis to give rise to the semicircular canal ducts and sensory chambers of the inner ear.

## Early establishment of neural competence and a neurogenic domain

During placodal development, a critical first step is the delineation of neurogenic versus non-neurogenic domains in the emerging placode. Several models for placode induction and acquisition of neurogenic competence have been discussed in the literature. One possibility is that induced placodes are inherently neurogenic. Alternatively, there may be a more general neurogenic inductive event, followed by restriction or independent specification of neurogenic and non-neurogenic fates. Interestingly, regardless of the sequence of events, similar underlying molecular programs are involved in both the otic and olfactory placodes (Fig. 1C).

### Olfactory

Olfactory placodal cells are specified at the gastrula stage (Hamburger and Hamilton stage 4 (HH4)). Explants isolated from the anterior border region give rise to cells of either lens or olfactory identity in distinct regions, while slightly later, at the neural fold stage (HH8), olfactory and lens placodal cells become spatially separated (Sjödal et al., 2007). Explants of presumptive olfactory tissue isolated from both gastrula and neurula stage embryos cultured in vitro give rise to neuronal cells of olfactory placodal identity which express markers for olfactory post-mitotic neurons (Sjödal et al., 2007; Maier and Gunhaga, 2009). This suggests that, once specified, presumptive olfactory placodal cells have neurogenic potential.

As development proceeds, presumptive olfactory placodal cells mature, and change in their responsiveness to BMP signaling. Whereas at early stages BMP induces prospective lens placodal character in olfactory placodal explants, at later stages, BMP signaling promotes respiratory epithelial non-neurogenic olfactory fates in chick and mouse (Sjödal et al., 2007; Maier et al., 2010). *Fgf8* is expressed anteriorly, and is required for the emergence of sensory olfactory epithelial cells. FGF signals also act to restrict the range of BMP activity in the nasal epithelium, limiting the extent of the respiratory epithelium (Maier et al., 2010). Thus, BMP signaling at later stages is required for the emergence of the non-neurogenic olfactory domain, while FGF signaling is required to maintain the neurogenic region. *Raldh3* expression overlaps with the *Fgf8* expression domain, whereas *Bmp4* is expressed posteriorly. This raises the interesting possibility that RA, FGF and BMP signaling act to subdivide the olfactory placode and regulate the coordinated emergence of neurons (Fig. 1C).

*Hes1* homologs are expressed in the future olfactory domain, where they act as prepatterning genes that define the neurogenic region. In addition, they play a later role in neurogenesis in mouse, chick and zebrafish (Cau et al., 2000; Thisse and Thisse, 2005; Maier and Gunhaga, 2009). These data suggest conserved functions of *Hes-*like genes during olfactory development throughout vertebrates. In addition to *Hes* genes, mutation of *Sox2* and its cofactor *Oct1* affects nasal development upstream of *Pax6* (Donner et al., 2007), suggesting a role for these factors in olfactory development.

### Otic

The otic placode becomes subdivided into an anterior neurogenic and posterior non-neurogenic domain. The neurogenic domain gives rise to the neurons of the VIIIth ganglion (statoacoustic ganglion, vestibuloacoustic ganglion, or vestibular and spiral ganglia, depending on species). This domain is likely to overlap with a broad zone of sensory competence that gives rise to the sensory hair cells in chick and mouse (Satoh and Fekete, 2005; Raft et al., 2007), since macular hair cells derive from a common *neurog1*-positive precursor that also gives rise to neurons (see below). In the fish, sensory hair cells arise at both the anterior and posterior poles of the otic vesicle (Haddon and Lewis, 1996). Although both sensory hair cell and neuronal lineages share many common regulatory features, recent data in zebrafish indicate that competence to form the neuronal lineage precedes that to form the sensory lineage (Hans et al., 2013). Manipulations in *dlx3b*/*4b* or *foxi1* reveal a role for these transcription factor genes in acquisition of sensory versus neuronal competence, respectively. This developmental decision occurs early, during induction of the otic-epibranchial precursor domain. In embryos carrying a homozygous deletion that removes *dlx3b*, *dlx4b* and *sox9a* genes (*b380* mutants), almost all otic fates, including the sensory lineage, are lost. Nevertheless, expression of otic neuroblast markers (*neurod*, *cdh6*) persists. Conversely, mutation of *foxi1* compromises otic neurogenesis, while sensory cells still form. Morpholino-mediated knockdown of *foxi1* in *b380* mutants results in the loss of both sensory and neuroblast fates in the ear (Hans et al., 2013). Thus, in zebrafish, otic neuronal competence is critically dependent on *foxi1* function, while *dlx3b/4b* genes promote sensory competence. It still remains to be elucidated whether a similar mechanism occurs in other species.

Sox3 and Sox2 have been implicated in acquisition of neural (both sensory and neuronal) competence downstream of FGF signaling (Abelló et al., 2010). Disruption of *Sox2* in mouse impairs formation of the sensory domain (Kiernan et al., 2005). Sox2 directly binds to the *Atoh1* promoter and activates its expression (Kiernan et al., 2005; Neves et al., 2012), acting in a feed-forward loop with other bHLH factors, and in co-operation with Six1, upstream of *Atoh1* (Ahmed et al., 2012; Neves et al., 2012). In addition, Sox2, possibly together with Sox3, drives neuronal differentiation in the chick ear (Neves et al., 2012) and may play a role in acquisition of otic sensory competence in the zebrafish (Sweet et al., 2011).

Tbx1, a T box transcription factor, acts to restrict the extent of the neurogenic domain in the otic vesicle: it is expressed in the non-neurogenic domain of the otic epithelium in mouse and zebrafish, and the neurogenic domain is expanded in *Tbx1* mutants in both species (Raft et al., 2004; Radosevic et al., 2011). In zebrafish, Tbx1 acts through the Hairy/Enhancer of Split (Hes) gene *her9*, which is co-expressed with *tbx1*. Morpholino-mediated knockdown of *her9* results in a similar expansion of the neurogenic domain (Radosevic et al., 2011). At least four additional *Hes*-like genes are expressed in the otic placode and vesicle in zebrafish (Thisse and Thisse, 2005; Gajewski et al., 2006), raising the possibility that *Hes*-like genes also might be involved in neurogenic patterning.

Expression of *Tbx1* in the ear is regulated by extrinsic signaling factors. The retinoic acid (RA) synthesizing enzyme gene *Raldh2* is expressed in the mesoderm surrounding the otic placode and is required to specify *Tbx1*-positive cells in zebrafish, chick and mouse (Bok et al., 2011; Radosevic et al., 2011). It is likely that RA regulates *Tbx1* expression directly during a narrow temporal window (Bok et al., 2011). By contrast, otic *tbx1* expression is repressed in zebrafish embryos with over-active Hedgehog (Hh) signaling (Hammond et al., 2010), and is induced by Hh inhibition (Radosevic et al., 2011). Thus, RA and Hh signaling appear to play a role in positioning the boundary between neurogenic and non-neurogenic domains in the ear.

BMP signaling also affects gene expression (*Lmx1b*) in the non-neurogenic domain of the otic placode/vesicle/cup, but has no effect on *Sox3* expression and neurogenic fate (Abelló et al., 2010). In chick, members of the Delta/Notch pathway are differentially expressed in the otic placode/cup: *Delta1, Hes5* and *Lunatic fringe* are restricted to the neurogenic domain, while *Serrate1* and *Hairy1* are expressed in the non-neurogenic domain (Abelló et al., 2007). Notch inhibition in neurula stage chick embryos causes expansion of the non-neurogenic domain and overproduction of neural precursors in the remaining neurogenic region (Abelló et al., 2007). Thus, Notch signaling is required to restrict the non-neurogenic domain. In zebrafish *mindbomb* mutants, in which Notch signaling is disrupted, increased numbers of neuroblasts and sensory hair cells develop in the ear (Haddon et al., 1998; Haddon et al., 1999).

FGF genes are also expressed around the otic placode/vesicle and later in the otic vesicle itself. Like BMPs, FGFs play important roles at multiple steps of otic development. In both chick and zebrafish, otic *Sox3* expression is dependent on FGF signaling (Nikaido et al., 2007; Sun et al., 2007; Abelló et al., 2010). In zebrafish embryos treated with the FGF inhibitor SU5402 after formation of the otic placode, expression of *neurod* in the developing otic ganglion is reduced, whereas ubiquitous over-expression of *fgf3* at the 10 somite (early placode) stage results in the duplication of a *neurod-*expressing domain at the posterior of the ear (Hammond and Whitfield, 2011). Taken together, these results demonstrate both the requirement and sufficiency of FGF for early otic neurogenic development, in addition to its roles in the neuronal (Alsina et al., 2004; Vemaraju et al., 2012) and sensory lineages (Millimaki et al., 2007; Sweet et al., 2011) at later stages.

In conclusion, the same signaling pathways regulate the early patterning events that lead to the generation of neurogenic and non-neurogenic territories, with some similarities and some differences between the olfactory and otic placodes (Fig. 1C). In both systems, FGF signaling promotes neurogenic character, whereas BMP promotes non-neurogenic character. RA, on the other hand, acts as a positive regulator of neurogenic fate in the olfactory placode, but acts as a negative regulator of neurogenic fate in the otic placode.

## Dual placode and neural crest contributions to otic and olfactory derivatives

The notion that placodes contribute exclusively to all cell types in mature sensory organs—with the known exceptions of peripheral glia of all sensory ganglia, and intermediate cells of the mammalian cochlea, which originate in the neural crest—has been challenged over the last few years. Recently, a neural crest contribution has been reported for both neuronal and non-neuronal components of the otic and olfactory systems (Dutton et al., 2009; Barraud et al., 2010; Forni et al., 2011; Freyer et al., 2011; Katoh et al., 2011; Saxena et al., 2013).

Occasional contribution of neural crest cells to the ear has been demonstrated in zebrafish embryos. Single-cell labeling of neural crest cells located at the level of rhombomere 4 revealed a small proportion of clones that contributed to the early otic vesicle, though most of these cells were lost by the third day of development (Dutton et al., 2009). Transgenic labeling studies in mouse, using driver lines expressing GFP in neuroepithelial cells, claim a more substantial neuroepithelial (and possibly neural crest) contribution to the inner ear and VIIIth ganglion (Freyer et al., 2011). However, these results remain controversial, given the recently raised problems with some of the Cre driver lines used for these studies (Lewis et al., 2013).

In zebrafish, a recent study provides support for the neural crest origin of microvillous sensory neurons within the olfactory epithelium using lineage analysis of early migrating neural crest cells via photoconversion (Saxena et al., 2013). Neural crest cells surround the olfactory capsule and a subpopulation intercalates into the olfactory epithelium, differentiating into microvillous neurons. Moreover, ablation of the neural crest caused a depletion of microvillous neurons in the nose but had only a minimal effect on the ciliated neurons, suggesting that placodal cells cannot compensate for the loss of neural crest-derived sensory neurons. Taken together, these studies indicate that neural crest cells may contribute to various lineages in the nose and inner ear of zebrafish. It is not clear whether this finding can be generalized to mammals; more detailed lineage studies are necessary to resolve this important question.

One additional cell type that is now considered to be a neural crest derivative in several species is the olfactory ensheathing cell. Olfactory ensheathing cells myelinate the olfactory nerve and are of great interest due to their clinical potential to promote axon regeneration in the CNS (Kawaja et al., 2009). Although it had been assumed that olfactory ensheathing cells derive from the olfactory placode (Couly and Le Douarin, 1985), fate mapping studies in chick and mouse recently have suggested that olfactory ensheathing cells are in fact neural crest-derived (Barraud et al., 2010; Forni et al., 2011; Katoh et al., 2011). In addition, cells of neural crest origin have been detected in the embryonic and postnatal olfactory epithelium, where they may contribute to horizontal basal cells, globose basal cells, sustentacular cells and Bowman glands/ducts (Katoh et al., 2011). Forni and colleagues (Forni et al., 2011) detected a similar range of neural crest-derived cell types as well as olfactory marker protein (OMP)-positive olfactory sensory neurons and a neural crest contribution to the vomeronasal organ. As mentioned above, the issues with many of the Cre lines used in the experiments mean that further confirmation will be required for these results.

In the chick ear, cochleo-vestibular glia derive from neural crest cells (D’Amico-Martel and Noden, 1983; Hemond and Morest, 1991) and develop in tandem with the otic sensory neurons to guide their projection to the CNS (Sandell et al., 2014).

## Neurogenesis in the olfactory and otic placodes

**Fig. 2:**
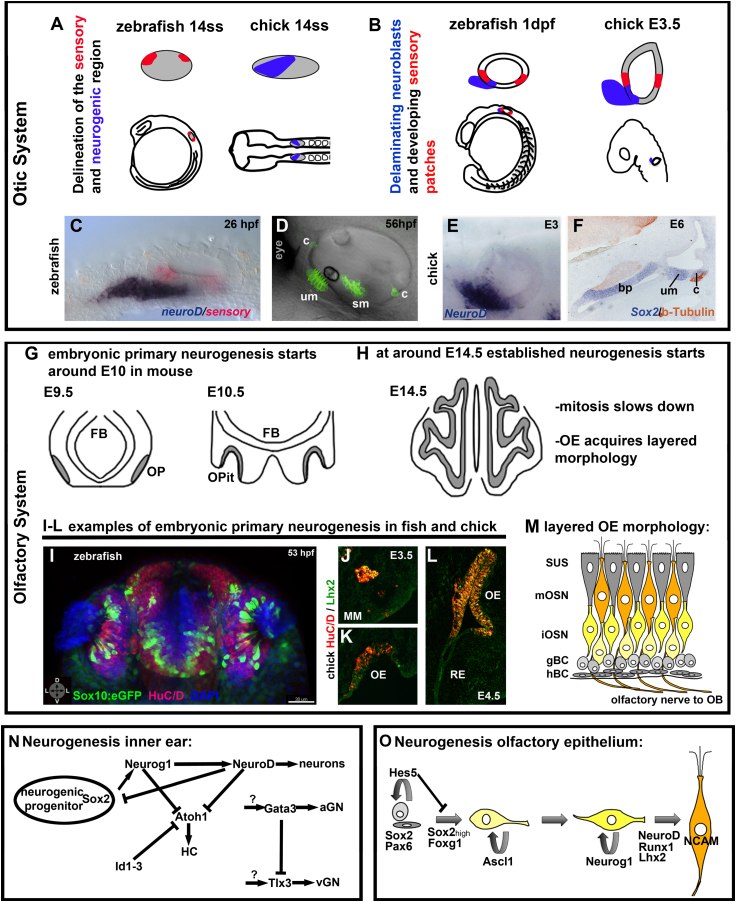
(A) Schematic drawing comparing the prospective neurogenic region in the otic placode from a 14 somite stage (ss) zebrafish embryo and a 14ss chick embryo. In the zebrafish, the sensory marker *atoh1b* is already expressed at 14ss (Millimaki et al., 2007), slightly ahead of the neurogenic marker *neurog1* at 16ss (Radosevic et al., 2011). By contrast, in the chick, expression of *ngn1* appears before the expression of sensory markers. (B) Schematic drawing depicting the delaminating neuroblasts and the developing sensory patches in the otic vesicle at comparable developmental stages in zebrafish and chick. (C) In situ hybridization showing neuroblasts (*neuroD*, blue) and sensory patches (red; unpublished sensory marker) in a 26 hours post fertilization (hpf) zebrafish otic vesicle. Lateral view; anterior to left. (D) Confocal *z*-stack projection of a *Tg(pou4f3:GFP)* zebrafish otic vesicle marking hair cells in the developing sensory patches at 56 hpf. Lateral view; anterior to top left, dorsal to top right. (E) In situ hybridization showing neuroblasts (*neuroD*, blue) in an E3 chick otic vesicle. Coronal section; anterior to the left, dorsal up. (F) In situ hybridization (*Sox2*, blue, marking sensory epithelia) and immunohistochemistry (β-Tubulin, brown, marking neuronal structures) in an E6 chick otic vesicle. Transverse section; medial to the left, dorsal up. (G) Schematic drawing depicting stages of olfactory placode development and primary neurogenesis in the mouse (coronal section). (H) Schematic drawing depicting the developmental stage that marks the transition from primary to established neurogenesis in mouse. (I) Example of primary neurogenesis in the zebrafish olfactory system. Confocal *z*-stack of antibody staining at 53 hpf: *Tg(–4.9sox10:eGFP)* (Wada et al., 2005; Carney et al., 2006) labels neural crest-derived microvillous neurons (Saxena et al., 2013), green; HuC/D labels post-mitotic neurons, red; nuclear stain, blue. Orientation arrows: D: dorsal; V: ventral; and L: lateral. Scale bar: 30 μm. (J–L) Examples of primary neurogenesis in the chick olfactory system. HuC/D labels post-mitotic neurons, red; Lhx2 labels progenitor cells and differentiated OSNs, green. (M) Schematic drawing depicting the layered morphology of the olfactory epithelium during established neurogenesis stages. (N) Scheme depicting the genetic network controlling neurogenesis in the inner ear. (O) Scheme depicting neurogenesis in the olfactory epithelium. Abbreviations: aGN: auditory ganglion neuron, bp: basilar papilla, c: crista, d: dorsal, dpf: days post fertilization, FB: forebrain, gBC: globose basal cell, HC: hair cell, hBC: horizontal basal cell, hpf: hours post fertilization, iOSN: immature olfactory sensory neuron, l: lateral, MM: migratory mass, mOSN: mature olfactory sensory neuron, OB: olfactory bulb, OE: olfactory epithelium, OP: olfactory placode, OPit: olfactory pit, RE: respiratory epithelium, sm: saccular macula, ss: somite stage; SUS: sustentacular cells, um: utricular macula, v: ventral, and vGN: vestibular ganglion neuron.

In the olfactory system, neurogenesis occurs throughout life such that there is a steady-state production of OSNs to replace damaged neurons. Two phases of olfactory neurogenesis have been described (Fig. 2G–M and O). First, primary neurogenesis occurs in the invaginating placode, establishing the basic structures of the olfactory epithelium and giving rise to a number of migratory neuronal cell populations that move to the olfactory bulb (Valverde et al., 1993; Drapkin and Silverman, 1999; Fornaro et al., 2003; Kawauchi et al., 2004; Maier and Gunhaga, 2009; Miller et al., 2010). Primary neurogenesis starts at approximately E10 in mouse and HH14 in chick (Kawauchi et al., 2005; Maier and Gunhaga, 2009) (Fig. 2G). A second wave of neurogenesis, termed established neurogenesis, occurs in the maturing pseudo-stratified epithelium (Fig. 2H). Here, stem cells give rise to transit amplifying neuronal progenitors, which in turn form immature neuronal precursors and finally give rise to OSNs (Calof et al., 2002).

Otic neurogenesis begins at the otic placode stage and proceeds until late stages of otic development (Fig. 2A–F and N). The duration of otic neurogenesis has not fully been determined, but in chick, neurogenesis proceeds at least until E6 (Bell et al., 2008). From E9 in mouse and 17 hours post fertilization (hpf) in zebrafish, neuroblasts begin to delaminate from the otic vesicle, migrate away and coalesce anteriorly to the otic vesicle to form the VIIIth ganglion (Carney and Silver, 1983; Haddon and Lewis, 1996; Vemaraju et al., 2012). In zebrafish, otic neuroblast specification and delamination peaks at around 24 hpf but continues until 42 hpf (Haddon and Lewis, 1996; Vemaraju et al., 2012). Proliferation continues in neuroblasts from the statoacoustic ganglia in a series of transit-amplifying divisions that expand the pool of neuronal progenitors (Alsina et al., 2003). In contrast to olfactory neurogenesis, no otic neurogenesis during adulthood has been reported in mammals to date.

### Intrinsic factors regulating neurogenesis

As cells progress from neural progenitor to mature neuron, distinct sets of transcription factors are expressed at different developmental times. Members of the Sox-B1-type SRY transcription factor family (Sox2) and of the bHLH transcription factor family (Neurog1) are expressed in most neurogenic tissues, including the otic and olfactory placodes (Fig. 2N and O).

#### Intrinsic factors regulating olfactory neurogenesis

During murine olfactory development, *Sox2* is expressed throughout the prospective neuroepithelium of the invaginating placode from E10.5 onwards, in a pattern similar to *Hes1/Hes5* (Cau et al., 2000; Kawauchi et al., 2005). *Sox2* is co-expressed with *Fgf8* at the rim of the invaginating placode. In *Fgf8* null mutant mice, these *Sox2*-expressing cells undergo apoptosis, and neurogenesis fails to occur (Kawauchi et al., 2005). Later (from E11.5 onwards), *Sox2* expression in the olfactory epithelium appears graded (high medial, low lateral). Some *Sox2*-positive cells co-express Ascl1 and mainly reside in the medial olfactory epithelium, where they divide rapidly (Tucker et al., 2010). Elevation of *Sox2* expression in lateral olfactory epithelium, where it is normally low, enhances neurogenesis. Thus, Sox2 functions as a dose-dependent regulator of precursor state in the olfactory epithelium. In the adult olfactory epithelium, *Pax6* and *Sox2* are expressed in basal cells and are upregulated before Ascl1 expression after damage (Shetty et al., 2005; Guo et al., 2010) (Fig. 2O).

During olfactory epithelial development, *Hes* genes have been implicated as pre-patterning genes that define the neurogenic domain within the olfactory placode at early stages (Cau et al., 2000; Maier and Gunhaga, 2009). Later, they control the number of neural progenitors by negatively regulating neurogenesis (Cau et al., 2000). Ascl1 is expressed in both the developing and established olfactory epithelium and is a marker for transient amplifying neuronal progenitors (DeHamer et al., 1994; Gordon et al., 1995; Cau et al., 2000; Calof et al., 2002; Beites et al., 2005). Loss of *Ascl1* function leads to a severe reduction of OSNs in the olfactory epithelium (Guillemot et al., 1993). Ascl1 acts upstream of *Neurogenin1* (*Neurog1*) and *NeuroD* in the OSN lineage and is required to initiate OSN differentiation (Cau et al., 1997). In the absence of Ascl1 function, progenitor cells fail to differentiate and levels of both proliferation and apoptosis in the olfactory epithelium increase, with surplus proliferating cells expressing markers of both neuronal progenitors (*Ascl1 3′UTR*) and supporting cells (*Steel*) (Murray et al., 2003). Ascl1-positive cells mature into *Neurog1-*expressing neurons. Accordingly, olfactory neuron differentiation is blocked in *Neurog1* null mutant mice (Cau et al., 2002). The Forkhead family member *Foxg1* is expressed in the developing mouse olfactory placode in a pattern similar to *Sox2* (Duggan et al., 2008). *Foxg1*-positive cells are located basally in the olfactory epithelium, and both mice and zebrafish lacking *Foxg1* have impaired differentiation of OSNs (Duggan et al., 2008; Kawauchi et al., 2009).

*NeuroD* is expressed late in *Neurog1*-positive progenitors when they start differentiating (Cau et al., 1997). After birth, *NeuroD*-positive cells in mice co-express *Runx1* (Theriault et al., 2005), which may act at the transition between immature neuronal precursor and OSN. *Runx1* is sufficient to expand the mitotic population in vitro (Theriault et al., 2005), making it a possible co-ordinator of proliferation and differentiation during olfactory neuron development. The LIM-homeobox gene *Lhx2* is expressed in both progenitor cells and olfactory neurons (Hirota and Mombaerts, 2004; Kolterud et al., 2004). In *Lhx2* null mutant mice, both Ascl1-positive and Neurog1-positive neuronal progenitors are normally distributed, but the expression of *Neurod1* and apoptosis markers is increased and expression of late differentiation markers is reduced (Hirota and Mombaerts, 2004; Kolterud et al., 2004).

#### Intrinsic factors regulating otic neurogenesis

The inner ear expresses members of the atonal family. Neurog1 is the earliest determination factor for the domain of neuronal precursors (Adam et al., 1998; Ma et al., 1998; Andermann et al., 2002). From this region, neuronal precursors are singled out by Notch-mediated lateral inhibition (Adam et al., 1998; Haddon et al., 1998). Cells expressing high levels of Neurog1/Delta1 become neuronal precursors, retaining expression of Sox2/3 and activating *Hes5* expression in their neighbors. High levels of Neurog1 or Delta1 activate other bHLH genes such as *NeuroD4/NeuroM* followed by *NeuroD1* (Adam et al., 1998; Kim et al., 2001; Bell et al., 2008). NeuroD1 has been shown to be essential for neuroblast delamination and survival (Kim et al., 2001) (Fig. 2N).

In the mouse, neuroblasts emigrate from a small domain of the otic vesicle that includes precursors of the utricular and saccular macula (Raft et al., 2007). Interestingly, neuroblasts exiting the epibranchial placodal epithelium in chick do not express characteristic epithelial-to-mesenchymal transition (EMT) markers such as *Snail2*, nor do they adopt a mesenchymal phenotype (Graham et al., 2007). It remains to be seen if this is a general feature of all placodal neurogenesis; however, the *snail2* gene is expressed in the neurogenic region of the zebrafish otic vesicle (Whitfield et al., 2002). Once they have migrated to the site of the VIIIth ganglion, otic neuroblasts express transcription factors such as Islet1 or POU4f1 (Huang et al., 2001; Radde-Gallwitz et al., 2004), together with the survival factor IGF1 (Camarero et al., 2001). In mouse, neuronal subtype identity (cochlear versus vestibular) correlates with the expression of the zinc finger transcription factor *Gata3*, which is initially expressed throughout the otic epithelium but becomes largely restricted to auditory neurons (Karis et al., 2001; Lawoko-Kerali et al., 2004; Jones and Warchol, 2009; Lu et al., 2011). Neurons are still generated in *Gata3* mutant mice, but a severe reduction in sensory cochlear neurons has been reported (Karis et al., 2001; Duncan and Fritzsch, 2013). The transcription factors Lmx1a and Tlx3 have been implicated in the regulation of vestibular neuron development. Lmx1a is co-expressed with Gata3 in neurogenic regions of the otic vesicle. Mice with disrupted *Lmx1a* (*dreher* mutants) have increased numbers of vestibular neurons; *Tlx3* is upregulated, but *Gata3* is not affected (Koo et al., 2009). Tlx3 is only expressed in vestibular neurons (Koo et al., 2009; Lu et al., 2011), but its precise function is currently unknown. Other factors, such as birth date, position and further signaling may determine whether the neurons innervate auditory or vestibular cells (Bell et al., 2008; Sapède and Pujades, 2010).

#### Intrinsic factors regulating otic sensorigenesis

Sensory hair cells are produced from sensory stem cells that express Sox2. The shared expression of Sox2 in the neurogenic and sensory stem cells may indicate a putative common progenitor for both hair cells and neurons. However, in chick, data indicate that otic neurons share common progenitors only with hair cells of the utricular macula (Satoh and Fekete, 2005). In mice, cell lineage tracing experiments using the *Neurog1* reporter transgenic mouse further support the interpretation that not all hair cells derive from a common progenitor with the neurons. Only hair cells of the utricular and saccular maculae derive from a progenitor expressing Neurog1 at early time points (Raft et al., 2007). Similarly, in zebrafish, a common progenitor giving rise to both sensory neurons and hair cells of the posterior macula has only been described in the posterior domain of the placode (Sapède et al., 2012). Choice of neuronal or sensory fate by the daughter cells of such a common progenitor might depend on the balance between the proneural factors *Neurog1* and *Atoh1*, which cross-regulate each other (Raft et al., 2007). Although the signals that promote the extinction of *Neurog1* expression together with an increase of *Atoh1* are still not well defined, Notch and BMP4 signaling pathways are likely to be key mediators of this process (Cole et al., 2000; Daudet and Lewis, 2005; Pujades et al., 2006; Kamaid et al., 2010; Jeon et al., 2011) (Fig. 2N).

Activation of the proneural gene *Atoh1* is indicative of sensory hair cell differentiation. Loss of *Atoh1* in mice or zebrafish results in loss of hair cells; conversely, its over-expression results in ectopic hair cell formation (Bermingham et al., 1999; Zheng and Gao, 2000; Chen et al., 2002; Woods et al., 2004; Millimaki et al., 2007; Liu et al., 2012; Xu et al., 2012; Yang et al., 2012). In chick, sensorigenesis (marked by the expression of *Atoh1*) lags behind neurogenesis (marked by the expression of *Neurog1*) by several days (Adam et al., 1998; Pujades et al., 2006). However, in zebrafish, *atoh1* expression precedes *neurog1* expression (Millimaki et al., 2007; Radosevic et al., 2011). Conditional deletion of *Atoh1* function at later stages of cochlear development revealed additional roles in hair cell survival and stereocilia bundle formation (Cai et al., 2013; Chonko et al., 2013). Interestingly, loss of *Atoh1* leads not only to a loss of hair cells but also supporting cells (Bermingham et al., 1999; Chen et al., 2002; Driver et al., 2013).

In contrast to its role during neurogenesis, Notch signaling has a dual function in sensorigenesis; it initially promotes a broad sensory fate by a lateral induction mechanism mediated by Jagged1 (Kiernan et al., 2001; Kiernan et al., 2006) and secondly selects hair cells from support cells in a classical lateral inhibition mechanism mediated by Delta1 (Haddon et al., 1998; Lanford et al., 1999; Daudet and Lewis, 2005; Daudet et al., 2007); reviewed in Neves et al. (2013).

### Extrinsic factors regulating neurogenesis

Extrinsic signaling molecules control the transition between the stem cell precursor and differentiated fate choice outlined above, together with the acquisition of new expression domains of intrinsic transcriptional regulators. In addition, extrinsic signaling events impact on proliferation, cell survival, cell death and—at later stages of olfactory neurogenesis—cell position in the epithelium.

#### Extrinsic factors regulating olfactory neurogenesis

During early olfactory development, FGF, BMP, RA and Notch signaling have all been implicated in the regulation of neurogenesis in the invaginating olfactory pit in mouse and chick (Kawauchi et al., 2005; Maier et al., 2010, 2011; Paschaki et al., 2013). These pathways are also implicated in the regulation of olfactory neurogenesis at later stages, both during the embryonic phase of established neurogenesis and postnatally.

At early stages, *Fgf8* is expressed in the rim of the invaginating olfactory placode, where it defines a morphogenetic center that is crucial for neurogenesis, cell proliferation and morphogenesis (Kawauchi et al., 2005; Maier, 2009; Maier et al., 2010). In the absence of FGF signaling, the olfactory pit fails to invaginate in both mouse and chick (Kawauchi et al., 2005; Maier et al., 2010), and the remaining cells are of respiratory olfactory character (Maier et al., 2010). In part, FGF acts to counteract posteriorly emanating BMP signaling, as pSmad1/5/8 levels are raised in the absence of FGF signaling both in vitro and in vivo (Maier et al., 2010). In mouse, Fgf18 starts to be expressed in the olfactory epithelium from around E12.5 (Kawauchi et al., 2005) while Fgf2 is expressed in the olfactory epithelium after birth (Hsu et al., 2001; Kawauchi et al., 2004). Addition of FGF2 to sensory epithelial cell cultures derived from late embryonic or mature olfactory epithelium stimulates transient amplifying cell division and the maintenance of stem cells (DeHamer et al., 1994).

In chick, *Bmp4* expression starts approximately in the middle of the olfactory pit and reaches all the way posteriorly (Maier, 2009). At these early stages, BMP signaling is critical for the development of the respiratory epithelium, but cells in the sensory part of the olfactory pit express scattered pSmad1/5/8, indicating that BMP signaling is active in prospective sensory cells (Maier, 2009). Loss of BMP signaling in vitro in a chick explant assay blocks primary neurogenesis and cells are Hes5-positive (Maier et al., 2010); Notch signaling, in turn, is required to maintain Hes5-positive progenitor cells in the olfactory epithelium (Maier et al., 2011). The BMP inhibitor Noggin strongly inhibits the formation of neuronal colonies in sensory epithelium dissected from E14.5 to E16.5 (Shou et al., 2000), while BMP ligands show both inhibitory and stimulatory effects on later neurogenesis (Shou et al., 1999, 2000). Another TGFβ family member expressed in the olfactory epithelium is GDF11 (Nakashima et al., 1999), which plays a role in the established phase of olfactory neurogenesis. Exogenous GDF11, as well as lack of the GDF11 inhibitor Follistatin, decrease proliferation of progenitor cells, thus negatively impacting on neurogenesis (Wu et al., 2003). Conversely, GDF11 null mutant mice have increased numbers of proliferating Neurog1-positive precursors, which ultimately give rise to OSNs. Nevertheless, loss of GDF11 is not sufficient to rescue the phenotype in *Follistatin* null mutant mice fully, suggesting that additional mechanisms are in place to account for the decrease in neuron numbers (Gokoffski et al., 2011). Both Sox2-positive and Ascl1-positive cells are increased in the olfactory epithelium of *ActivinβB* null mice (Wu et al., 2003; Gokoffski et al., 2011), affecting the cell fate decision between glial and neuronal lineages (Gokoffski et al., 2011). Taken together, these results suggest that TGFβ signaling is crucial for feedback regulation of progenitor cell populations and fate choices in the olfactory epithelium.

RA emanating from the *Raldh3*-expressing anterolateral rim of the olfactory pit has been implicated in olfactory epithelium patterning and/or differentiation (LaMantia et al., 2000; Bhasin et al., 2003; Paschaki et al., 2013). In the absence of RA signaling, Pax6-positive cells are reduced in the olfactory epithelium while elevated levels of RA suppress neurite outgrowth in vitro (Paschaki et al., 2013). RA production in the olfactory epithelium persists during late embryonic development into adulthood. In adult mice, all three retinoic acid-producing enzymes (Raldh1, 2 and 3) are expressed in the olfactory epithelium (Niederreither et al., 2002; Peluso et al., 2012). Raldh3 expression is detected in a small population of basal cells in the ventral and lateral epithelium; gain- and loss-of-function data support the idea that RA signaling is involved in maintenance of the epithelium and OSN regeneration after injury (Peluso et al., 2012).

#### Extrinsic factors regulating otic neurogenesis

Several studies suggest a role for FGF signaling in otic neurogenesis in different species (Mansour et al., 1993; Hossain et al., 1996; Adamska et al., 2000; Pirvola et al., 2000; Adamska et al., 2001; Léger and Brand, 2002; Alsina et al., 2004; Millimaki et al., 2007) but analysis has been complicated due to overall defects in inner ear development. In zebrafish, *fgf3* and *fgf8* are expressed in and around the anterior otic vesicle and moderate levels of FGF signaling are required for neuroblast specification (Vemaraju et al., 2012). Mature neurons in the VIIIth ganglion start to express *fgf5*, with ongoing FGF signaling in the VIIIth ganglion serving two functions: firstly to delay differentiation of precursor cells, thus controlling precursor cell maintenance and the production of mature neurons, and secondly to terminate neuroblast specification when a sufficient number of *fgf5*-positive cells has accumulated (Vemaraju et al., 2012). In mammals, ongoing expression of *Fgf10* (Pirvola et al., 2000) might have similar functions in regulating the balance between transit amplifying neuroblasts and differentiating neurons. Thus differential FGF signaling regulates distinct phases of otic neurogenesis.

Mutations in the *insulin-like growth factor* (*Igf*) gene lead to hearing loss in both mice and humans (Camarero et al., 2001; Cediel et al., 2006; Walenkamp and Wit, 2007; Riquelme et al., 2010). Both *Igf* and its receptor are expressed in the VIIIth ganglion in chick and in vitro analysis revealed its role in VIIIth ganglion neuron survival, proliferation and differentiation (Camarero et al., 2003). Activation of the PI3–AKT pathway by IGF1 in proliferative neuroblasts is essential for neuroblast survival (Aburto et al., 2012). Other growth factors with a role in otic ganglion neuron proliferation, survival and innervation include BMP7, BMP4 and Shh (Fantetti and Fekete, 2012). The data for BMP signaling are sparse, but BMPs can promote neurite outgrowth and neuron survival in a chick in vitro system (Fantetti and Fekete, 2012).

Sensory cells secrete nerve chemoattractant molecules that influence axon routing. Some of the candidates for exerting a chemoattractant function in the inner ear are the neurotrophins, BDNF and NT3. Both are expressed in sensory patches, while VIIIth ganglion neurons express TrkB and TrkC receptors for BDNF and NT3, respectively (Fariñas et al., 2001). Ectopic expression of BDNF causes rerouting of axons (Tessarollo et al., 2004). In addition, VIIIth ganglion neurons express neuropilin/plexin receptor complexes while the otocyst expresses the repellent semaphorin3A. This prevents the sensory afferent axons from innervating inappropriate locations (Gu et al., 2003; Katayama et al., 2013). Recently, the EphB2 and EphA4 receptors have been shown to regulate the segregation of axonal entries at the brainstem in a cell-autonomous manner (Allen-Sharpley et al., 2013).

#### Extrinsic factors regulating otic sensorigenesis

Several signaling pathways have been implicated in hair cell formation in the inner ear. Shh is expressed in the developing spiral ganglion and has been shown to inhibit hair cell formation in cochlear explants (Driver et al., 2008; Liu et al., 2010). Shh signaling from the spiral ganglion regulates the time point at which hair cell precursors exit the cell cycle and differentiate (Bok et al., 2013; Tateya et al., 2013). In zebrafish, Shh signaling is required for the formation of hair cells from the saccular macula (Sapède and Pujades, 2010). Together with studies that show a requirement for Shh signaling in the development of auditory structures from the ventral otocyst, this has led to the speculation that Shh signaling was recruited during inner ear evolution to direct the formation of a second sensory patch with distinct auditory function (Riccomagno et al., 2002; Bok et al., 2005; Sapède and Pujades, 2010).

Wnt signaling is implicated at many stages of otic development. The Wnt pathway components are expressed in the chick basilar papilla (Sienknecht and Fekete, 2008) and ectopic activation of the canonical Wnt pathway leads to the formation of ectopic hair cells in chick non-sensory regions (Stevens et al., 2003). Using in vitro and in vivo approaches, a role for Wnt signaling in regulating sensory precursor cell proliferation and hair cell differentiation in the basilar papilla has been suggested (Jacques et al., in press). A similar role has been observed for hair cells in the zebrafish lateral line (Jacques et al., in press).

Both Notch and BMP4 signaling pathways have been implicated in sensory hair cell development (Cole et al., 2000; Daudet and Lewis, 2005; Pujades et al., 2006; Kamaid et al., 2010; Jeon et al., 2011). Expression of *Bmp4* marks all the emerging pro-sensory regions in mouse and most in zebrafish (Wu and Oh, 1996; Morsli et al., 1998; Cole et al., 2000; Mowbray et al., 2001). In vitro treatment of the chick otocyst with BMP4 leads to a decrease in the number of Atoh1-positive cells, while treatment with the BMP antagonist Noggin leads to an increase (Pujades et al., 2006). Increased BMP activity upregulates the expression of *Id1-3*, and the forced expression of Id1-3 is sufficient to reduce *Atoh1* expression and to prevent hair cell differentiation (Kamaid et al., 2010).

## Neuronal cell types and differentiation in the olfactory and otic systems

**Fig. 3:**
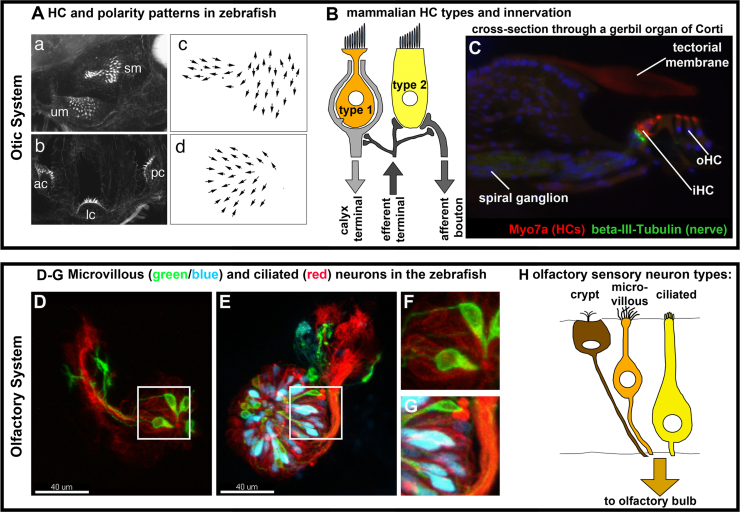
(A) Phalloidin stain and hair cell polarity patterns in zebrafish otic sensory patches at 3–4 days post fertilization: (a) utricular and saccular maculae, (b) cristae, (c) polarity pattern of the saccular macula and (d) polarity pattern of the utricular macula. Anterior to the left in all panels. Arrows in (c) and (d) are drawn from the stereociliary bundle to the kinocilium. Reproduced, with permission, from Hammond and Whitfield (2011). (B) Schematic drawing of the two types of mammalian vestibular hair cells and their innervation patterns. (C) Cross section through a gerbil organ of Corti. Myo7A labels hair cells, red; βIII Tubulin labels the spiral ganglion and nerve fibers, green; nuclei are labeled in blue. (D–G) Examples of olfactory cell types in live zebrafish at 29 hpf (D, F) and 60 hpf (E,G). Panels F and G show enlargements of the regions boxed in D and E, respectively. Confocal *z*-stacks of live embryos: *Tg(TRPC24.5k:gap-Venus)/rw037* (Sato et al., 2005) labels all microvillous neurons, green; *Tg(OMP2k:lyn-mRFP)/rw035* (Sato et al., 2005) labels all ciliated neurons, red; *Tg(–4.9sox10:eGFP)* (Wada et al., 2005; Carney et al., 2006) labels neural crest-derived microvillous neurons (Saxena et al., 2013), blue. Scale bar: 40 μm. (H) Scheme depicting sensory cells in the nasal epithelium. Abbreviations: ac: anterior crista, HC: hair cell, iHC: inner hair cell, lc: lateral crista, oHC: outer hair cell, pc: posterior crista, sm: saccular macula, and um: utricular macula.

The final outcome of neurogenesis in the ear and the nose is a wide variety of neurons and mechanosensory cells, exquisitely tailored for the detection of different sensory inputs. These differ significantly between the olfactory and otic placodes and are described below, and summarized in Fig. 3.

### Olfactory sensory neurons

The olfactory epithelium acquires a layered morphology and is populated by three broad classes of OSNs: ciliated, microvillous, and crypt neurons, with the first two being the dominant subtypes and the third present only in some species, including teleosts (Fig. 3D–H). OSNs can differ within subtypes, and, to a significant extent, across species. In general, the basally located ciliated sensory neurons, the most well-characterized of the OSNs, are responsible for volatile odorant detection, whereas the more apically located (and/or separately located in the vomeronasal organ) microvillous sensory neurons detect pheromones, nucleotides, amino acids and other molecules, depending on the species (Buck, 2000; Sato and Suzuki, 2001; Hansen and Zielinski, 2005). The crypt cells are located at the apical surface and have both microvilli and cilia (Hansen and Zeiske, 1998). Recent work in zebrafish has described the unusual relative homogeneity of these cells, wherein they all express an identical olfactory receptor and target a single glomerulus (Gayoso et al., 2012; Oka et al., 2012; Ahuja et al., 2013).

In addition to OSNs, populations of migratory neuronal cells leave the olfactory placode/pit and move towards the forebrain. Collectively, the heterogeneous populations of migratory cells and the extending OSN axons are termed the “migratory mass”. A first wave of migratory neuronal cells (GnRH-negative) is seen directly after onset of neuronal differentiation in the olfactory placode, before OSN axons have emerged from the olfactory placode: these early migratory cells are thought to play a role in establishing a scaffold for the extension of OSN axons slightly later (Croucher and Tickle, 1989; De Carlos et al., 1995; Maier and Gunhaga, 2009) (Fig. 2J). Later migratory cell populations along the forming olfactory nerve include the GnRH-positive neuronal cells that migrate along the olfactory nerve to the hypothalamus and the nervus terminalis (Schwanzel-Fukuda and Pfaff, 1989; Wray et al., 1989a, 1989b); (reviewed in Cariboni et al. (2007)), the olfactory ensheathing glia, OMP-positive cells and acetylcholine esterase-positive cells (De Carlos et al., 1995; Miller et al., 2010). Neuroactivity in the olfactory system is modulated both in the olfactory bulb and the olfactory epithelium. The best-described modulatory pathway is via the terminal nerve and GnRH, but other neuropeptides are also present (reviewed in Whitlock (2004), Kawai et al. (2009)).

In mice, the picture is further complicated by the presence of a variety of different microvillous cells in the main olfactory epithelium that may or may not be neuronal in nature. It has been suggested that secondary sensory and/or non-neuronal microvillous cells, possibly in co-operation with ciliated neurons, are involved in chemosensation. Various subtypes and possible roles have been identified to date in multiple publications, often with conflicting conclusions (Rowley et al., 1989; Asan and Drenckhahn, 2005; Elsaesser et al., 2005; Elsaesser and Paysan, 2007; Hansen and Finger, 2008; Lin et al., 2008; Hegg et al., 2010; Ogura et al., 2011). As such, more work is required to determine comprehensively how many subtypes of microvillous cells exist in the mammalian olfactory epithelium, whether any of these have direct neuronal sensory roles, and what proportion have secondary sensory properties as opposed to solely supporting roles. These microvillous cells are reminiscent of the integrated ciliated/microvillous architecture seen in teleosts, and add further to the debate regarding the presence and mechanisms of pheromone-sensing/vomeronasal OSNs in humans.

In addition to cell type, the OSNs vary with regard to odorant receptor expression. Odorant receptors are the largest family of mammalian genes, with ~380 genes in humans and ~1280 genes in rodents (Buck and Axel, 1991; Zhang and Firestein, 2009), and odor discrimination requires the differential expression of these genes. The OSNs are almost always uniquely specified to express only one odorant receptor (Chess et al., 1994; Malnic et al., 1999). The expression of a single odorant receptor per OSN is achieved by both random and non-random processes: mono-allelic expression (random) (Chess et al., 1994), zonality (four zones, non-random) (Ressler et al., 1993; Vassar et al., 1993; Sullivan et al., 1995; Qasba and Reed, 1998; Norlin et al., 2001; Zhang et al., 2004), initial odorant receptor selection, switching (random) (Shykind et al., 2004) and feedback by the expressed odorant receptors (non-random) (Serizawa et al., 2003). *Msx1*, *Alk6*, *Raldh2* and *Neuropilin* are expressed in the adult mouse olfactory epithelium in patterns correlating with the zonal distribution of odorant receptors (Norlin et al., 2001), but no functional role for zonal specification has been described so far. In addition, homeodomain binding sites are found in odorant receptor promoter regions and several homeodomain proteins have been shown to bind an odorant receptor promoter in vitro (Hoppe et al., 2003; Hirota and Mombaerts, 2004). Both Emx2 and Lhx2 are implicated in odorant receptor choice (Hoppe et al., 2003; Hirota and Mombaerts, 2004; Kolterud et al., 2004; Hirota et al., 2007), and odorant receptor expression frequency is altered in Emx2 null mice (McIntyre et al., 2008). Additional enhancer elements (H and P elements) also influence odorant receptor frequency (Serizawa et al., 2003; Bozza et al., 2009; Khan et al., 2011).

### Otic sensory hair cells

Hair cells are the sensory unit of the inner ear. The hair cell bundle is composed of actin-rich stereocilia (a type of microvilli) that are linked to each other by a series of connecting links (tip links, top connectors and ankle links). Protein constituents of the tip link include Protocadherin15, Cadherin23 and Harmonin, which together act to transduce the mechanical force to the anion mechanotransducer channel (reviewed in Schwander et al. (2010), Richardson et al. (2011), Kazmierczak and Müller (2012)). The hair bundle is polarized in the plane of the epithelium, with the kinocilium positioned on one side of the hair bundle, nearest the tallest stereocilia, and connected to it by kinociliary links. This arrangement gives the hair cell its directional sensitivity, with maximum stimulation occurring when the stereociliary bundle is deflected in the direction of the kinocilium. Examples of polarity patterns in the zebrafish ear are shown in Fig. 3A.

Vestibular epithelia in the amniote ear contain both Type I and II vestibular hair cells (Fig. 3B), which differ in their electrophysiological properties and synaptic contacts with afferent endings of the VIIIth nerve (Wersäll, 1956; Correia and Lang, 1990; Lysakowski and Goldberg, 1997; Eatock et al., 1998; Moravec and Peterson, 2004). In fish and amphibians, only type II vestibular hair cells occur, which are contacted by bouton afferent endings (Wersäll, 1956; Goldberg et al., 1990). Recent work in zebrafish, however, is beginning to uncover significant variation in both morphology and electrophysiological properties of hair cells from different regions of the ear (Haden et al., 2013). While both Wnt and Shh signaling have been implicated in the ectopic acquisition of vestibular identity (Stevens et al., 2003; Driver et al., 2008), little is known about the differentiation of the various types of sensory cells in the vestibular sensory epithelia.

In amniotes, the exquisite spatial organization of hair cells along the auditory epithelium is critical for the discrimination of auditory stimuli at different frequencies. This is particularly advanced in the mammalian cochlea, where orderly rows of hair cells are organized in a tonotopic array in the organ of Corti. One row of inner hair cells and three rows of outer hair cells run along the cochlear duct from the base (high frequency detection) to the apex (low frequency detection) (reviewed in Raphael and Altschuler (2003); Fig. 3C). Vibration of the basilar membrane, amplified by the outer hair cells, determines a specific position where sound vibration peaks (reviewed in Dallos et al. (2006), Dallos (2008)). It is the inner hair cells that convert this sound energy into an electrical signal: mechanical stimulation of the inner hair cells by their movement relative to the tectorial membrane results in depolarization and stimulation of afferent neurons of the spiral ganglion. In general, short stereociliary bundles are associated with high frequency detection, while long stereociliary bundles are associated with low frequency detection.

Birds have two types of hair cells in the basilar papilla, tall and short hair cells (Fischer, 1994). The tall hair cells are the equivalent of the inner hair cells in mammals, and are innervated by both afferent and efferent fibers. The short hair cells, by contrast, have no afferent innervation at all, but receive efferent input. It is possible that they have a modulatory role, but no evidence exits to date for this (reviewed in Köppl ( 2011)). A similar pattern is observed in the basilar papilla of lizards (Chiappe et al., 2007; Manley, 2011).

Distinct signals mediate differentiation of the lateral outer hair cell compartment versus the medial inner hair cell compartment of the developing cochlea, with evidence implicating Emx2, Delta-Notch and FGF signaling in this process. For example, *Emx2* null mutant mice have about 60% fewer hair cells than their wild-type littermates; the remaining hair cells label positive for Myo7 and *Fgf8*, two inner hair cell markers. Outer hair cells are completely lost, leading to the idea that Emx2 may regulate the balance between proliferation and differentiation in hair cell progenitors (Holley et al., 2010). Conditional loss of *Jag1* in the cochlea shifts the balance between outer and inner hair cells in favor of inner hair cells, consistent with the interpretation that Jag1 expands the prosensory state in the sensory epithelium via lateral induction; in addition, it regulates cell cycle exit via the expression of *p27*^*Kip1*^ (Brooker et al., 2006; Kiernan et al., 2006). FGF signaling is also important for hair cell development; Fgf20 is required in the lateral part of the cochlea, and *Fgf20* null mice are deaf and lack outer hair cells (Huh et al., 2012). After conditional loss of *Fgfr1*, hair cell precursors are reduced and preferentially differentiate into inner hair cells and pillar cells (Pirvola et al., 2002).

Mutations in Srrm4 (Deol, 1981; Whitlon et al., 1996; Nakano et al., 2012) and in a ‘self-terminating’ conditional Atoh1 allele (Pan et al., 2012) both predominantly lose inner hair cells. It has been proposed that Srrm4 might act downstream of Atoh1 in a feedback loop (Nakano et al., 2012). At later developmental stages, Atoh1 is expressed in inner hair cells earlier and at higher levels compared to outer hair cells and recent evidence indicates that prolonged expression is required for the correct hair cell differentiation, and that inner hair cells critically depend on Atoh1 for maintenance (Pan et al., 2012).

### Neurons of the VIIIth ganglion

Different types of afferent ganglion neurons innervate the sensory hair cells of the vestibular and auditory systems. In the vestibular system, afferent fibers ending in both bouton- and calyx-type synapses are found, depending on the species (Wersäll, 1956; Goldberg et al., 1990) (Fig. 3B). In mammals, most afferents are dimorphic, terminating in both bouton and calyceal endings, and contacting both Type I and Type II vestibular hair cells (Fernandez et al., 1990; Lysakowski et al., 1995). In the auditory system of mammals, there are two types of spiral ganglion cells, Type 1 and Type 2, both of which have bouton-type endings. Type 1 spiral ganglion cells contact inner hair cells and are large, bipolar cells that make up 90–95% of all the neurons in the spiral ganglion, in line with inner hair cells being the sensory receptor in the cochlea. Inner hair cells contact afferent endings from Type 1 ganglion cells at specialized ribbon synapses. Type 2 spiral ganglion cells contact outer hair cells and are small, bipolar or pseudomonopolar, and are less myelinated than Type 1 ganglion cells. Ribbon synapses with Type 2 cells occur, but only in the apical turn of the cochlea (reviewed in Raphael and Altschuler (2003), Nayagam et al. (2011), Bulankina and Moser (2012)).

## Neurodegeneration and regeneration

The sensory cells in the inner ear and nose in mammals differ greatly in their regenerative potential. The OSNs of the nose are routinely replaced throughout life. The average life span of a murine OSN is 90 days (Mackay-Sim and Kittel, 1991), but these cells may survive up to a year (Hinds et al., 1984). By contrast, no epithelial maintenance has been described for the hair cells of the cochlea of mammals, though hair cell addition and repair occurs in lower vertebrates (reviewed in Burns and Corwin (2013), Rubel et al.(2013)).

Whereas the OSNs are exposed to the outside world and regularly damaged, the hair cells of the inner ear are in a relatively protected environment. Nevertheless, sensory hair cells of the ear are lost through aging, and are also sensitive to damage from both noise and chemical exposure. Examples of hair cell-damaging drugs (ototoxins) include the aminoglycoside antibiotics, which are effective in the treatment of life-threatening infections, but can leave a patient deaf as a result. In mammals, lost auditory hair cells are not replaced; the reasons for this lack of regenerative potential are not fully understood. Often, once hair cells are damaged, the neurons of the VIIIth ganglion lose afferents and also undergo cell death with time (Webster and Webster, 1981; Schuknecht and Gacek, 1993).

### Regeneration in the adult olfactory system

The loss of the sense of smell—anosmia—can significantly impair quality of life and is often associated with trauma or neurodegenerative disease such as Parkinson’s or Alzheimer’s disease, in addition to normal aging (Doty et al., 1984; Doty, 2012). This sensory deterioration is all the more striking given the well-documented self-renewal of the olfactory epithelium throughout adulthood in healthy individuals. OSNs have the remarkable ability to regenerate in adult organisms, including in humans, and the basally located and horizontal basal cells are thought to be the predominant and likely sole source of all cell types in the regenerating olfactory epithelium post-injury (Graziadei and Graziadei, 1979; Leung et al., 2007; Iwai et al., 2008; Bermingham-McDonogh and Reh, 2011). Intriguingly, a recent study in mice using P0-Cre-based fate mapping has suggested that as the olfactory epithelium matures, placode-derived basal cells are slowly replaced by neural crest-derived basal cells (Suzuki et al., 2013). As the use of Cre-based fate mapping of neural crest is often fraught with non-neural crest-specific labeling, confirmatory studies will be needed to determine the validity of this striking finding.

The transcription factor p63 has been implicated in maintenance of the horizontal basal cell population in the adult olfactory epithelium, and both expression and mutant studies suggest that its down-regulation may play an important role in the differentiation of these cells in adults (Fletcher et al., 2011; Packard et al., 2011). In addition, retinoic acid signaling is critical for progenitor cell maintenance as well as for neurogenesis in the olfactory epithelium (Peluso et al., 2012; Paschaki et al., 2013). Indicating possible crosstalk between varied cell types, a report by Jia and colleagues suggests that inositol triphosphate receptor subtype 3- and neurotrophic factor neuropeptide Y-expressing microvillous cells play an essential role in olfactory neuron regeneration post-simulated injury (Jia et al., 2013).

### Regeneration of hair cells in the auditory and vestibular epithelia of the inner ear

Inner ear hair cells are produced throughout life in bony and cartilaginous fish (Corwin, 1981; Corwin, 1983; Popper and Hoxter, 1984; Lombarte et al., 1993; Bang et al., 2001; Smith et al., 2006; Schuck and Smith, 2009; Liang et al., 2012), and fish, amphibians and lizards are all capable of hair cell regeneration (Avallone et al., 2003; Taylor and Forge, 2005; Liang et al., 2012). In chick, hair cell number is restored in the basilar papilla exposed to either acoustic trauma (Cotanche, 1987), ototoxic drug exposure (Cruz et al., 1987; Kaiser et al., 2009) or basilar papillar lesion (Irvine et al., 2009). Nevertheless, no new production of hair cells occurs in the absence of damage, although vestibular epithelia produce new hair cells throughout life in birds (Cruz et al., 1987; Jørgensen and Mathiesen, 1988; Girod et al., 1989; Raphael, 1992; Roberson et al., 1992; Weisleder and Rubel, 1992, 1993; Kaiser et al., 2009).

After damage, supporting cells in the basilar papilla re-enter the cell cycle and eventually differentiate into supporting cells and hair cells in both neonatal chicken and adult birds (Corwin and Cotanche, 1988; Ryals and Rubel, 1988; Hashino et al., 1992; Raphael, 1992; Stone and Cotanche, 1994; Warchol and Corwin, 1996; Stone et al., 1999). In addition, there is direct transdifferentiation of supporting cells and subsequent compensatory proliferation in amphibian sacculi and avian basilar papillae in response to damage (Adler and Raphael, 1996; Baird et al., 1996; Adler et al., 1997; Steyger et al., 1997; Baird et al., 2000; Roberson et al., 2004). Accordingly, Atoh1a is expressed in supporting cells shortly after hair cell damage (Cafaro et al., 2007; Lewis et al., 2012). Whereas direct transdifferentiation occurs within a matter of hours, it takes days for hair cells to arise from mitotic division of supporting cells, which eventually comprise 50–70% of the regenerated hair cells (Roberson et al., 2004; Cafaro et al., 2007). These data suggest that direct transdifferentiation is a fast way to replace hair cells, while cell cycle re-entry is slower, but better suited to replace large numbers of hair cells (Stone and Cotanche, 2007). Similarly, in the lateral line of fish and amphibians, supporting cells have been shown to replace damaged hair cells (Balak et al., 1990; Ma et al., 2008) and hair cell regeneration by both transdifferentiation and proliferation has been suggested in the adult zebrafish inner ear (Schuck and Smith, 2009; Sun et al., 2011; Liang et al., 2012).

In birds, regenerative proliferation is likely to depend on chromatin modification, as inhibitors of histone deacetylation have been shown to prevent cell cycle entry and thus diminish regenerative proliferation (Slattery et al., 2009). In addition, screens to identify transcription factors expressed differentially during avian hair cell regeneration have shed light on potential signaling pathways involved in regeneration (Hawkins et al., 2007) and their role in regenerative proliferation (Alvarado et al., 2011).

After damage in mammals, by contrast, no new hair cells are formed in auditory sensory epithelia (organ of Corti) and supporting cells remain mitotically quiescent (Roberson and Rubel, 1994; Sobkowicz et al., 1997; Yamasoba and Kondo, 2006). While cochlear hair cells can be replaced in embryonic mice after laser ablation, neonatal mice lose this ability 5 days after birth (Kelley et al., 1995; Burns et al., 2012). The mammalian vestibular epithelium is slightly more plastic, with some cells transdifferentiating into immature hair cells (Forge et al., 1993, 1998; Warchol et al., 1993; Forge et al.), though full regeneration is rare (Warchol et al., 1993; Rubel et al., 1995; Li and Forge, 1997; Kuntz and Oesterle, 1998; Ogata et al., 1999; Oesterle et al., 2003). Nevertheless, hair cell generation has been induced in neonatal/postnatal mice by either manipulating the Notch or Wnt pathway (Yamamoto et al., 2006; Shi et al., 2012; Mizutari et al., 2013; Shi et al., 2013) and advances have been made to stimulate non-otic stem cells to differentiate into sensory hair cells (Boddy et al., 2012; Duran Alonso et al., 2012; Hu et al., 2012; Lin et al., 2012; Ouji et al., 2012).

Two major differences have emerged between supporting cells in the basilar papilla in birds and their counterparts in the mammalian cochlea. After damage in birds, but not mammals (Shailam et al., 1999; Batts et al., 2009), Atoh1 expression is detectable in actively dividing supporting cells in the basilar papilla (Cafaro et al., 2007). Misexpression of Atoh1 in the inner ear of developing or mature mammals induces hair cell characteristics in these cells (Zheng and Gao, 2000; Kawamoto et al., 2003; Shou et al., 2003; Izumikawa et al., 2005; Staecker et al., 2007), but this potential is lost with age (Kelly et al., 2012; Liu et al., 2012). Thus regulatory events upstream of Atoh1 or modifications on the level of the *Atoh1* promoter/chromatin structure may have altered mammalian otic sensory epithelia.

Another striking observation is that mammalian supporting cells have a decreased capacity for shape change (Forge, 1985; Cotanche, 1987). In the basilar papilla in birds, cell shape changes occur before cell cycle re-entry and cells adopt mature shapes once they differentiate into hair cells or support cells (Corwin and Cotanche, 1988; Marsh et al., 1990). This ability to undergo shape changes may be a requirement for regeneration. As mammalian supporting cells age, thick F-actin/E-cadherin belts are established in the apical junctions between supporting cells (Burns et al., 2008; Collado et al., 2011). By contrast, the supporting cells in chick, bony and cartilaginous fish, amphibians and turtles all have thin actin belts similar to those developing and neonate mice (Burns et al., 2013). However, it is currently unclear if this is permissive for regeneration or simply an interesting correlation. To date, the best approach for hair cell regeneration in zebrafish, and to a lesser extent in adult mammalian sensory epithelia, relies on Notch inhibition (Ma et al., 2008; Lin et al., 2011b; Mizutari et al., 2013).

### Regeneration of auditory neurons in the VIIIth ganglion

While auditory neurons strongly depend on neurotrophic input from their sensory targets and usually die after deafferentiation in mammals (Shi and Edge, 2013), re-innervation occurs routinely in species with regenerating hair cells. After hair cell damage, the dendrites retract and then grow out, make contact with a regenerated hair cell and form synapses (Ryals et al., 1992; Ryals and Westbrook, 1994; Ofsie and Cotanche, 1996; Hennig and Cotanche, 1998; Zakir and Dickman, 2006). Regeneration after statoacoustic nerve lesion has been reported in fish and amphibians (Newman et al., 1989; Mensinger and Highstein, 1999; Goto et al., 2002).

Re-innervation has been reported in several damage models, but is not necessarily correlated with functional recovery and depends on the severity of the induced damage (Johnsson and Hawkins, 1972; Terayama et al., 1977; Terayama et al., 1979; Webster and Webster, 1982; Bohne and Harding, 1992; Strominger et al., 1995; Lawner et al., 1997). In mouse and guinea pig models of neuronal hearing loss, no evidence for regenerated fibers and newly formed synapses was found (Kujawa and Liberman, 2009; Lin et al., 2011a; Yuan et al., 2014). Re-innervation (Martinez-Monedero et al., 2006) and synapse regeneration has recently been reported in a rat and mouse in vitro model (Wang and Green, 2011; Tong et al., 2013) and addition of Neurotrophin-3, BDNF or block of the repulsive guidance molecule RGMa increased synapse formation (Wang and Green, 2011; Brugeaud et al., 2013; Tong et al., 2013). Many advances have been made in trying to replace lost auditory neurons. Both inner ear cells and cells from other sources have successfully been stimulated in vitro to differentiate into neuronal cells (Li et al., 2003; Oshima et al., 2007) and to innervate hair cells in vitro (Kim et al., 2005; Shi et al., 2007; Martinez-Monedero et al., 2008; Matsumoto et al., 2008) and in vivo (Hu et al., 2005; Okano et al., 2005; Corrales et al., 2006; Coleman et al., 2007). Most promisingly, some functional recovery has been reported in a deafened gerbil model following transplantation of human embryonic stem cell-derived auditory neurons (Chen et al., 2012).

## Conclusion

Continued assembly of the molecular cascades responsible for neurogenesis in the olfactory and otic systems has yielded regulatory networks that are increasingly elaborate in scope and complexity. Knowledge and understanding of such networks is already driving forward progress towards the development of cell-based therapies for sensory disorders such as deafness. The recapitulation of otic development in three-dimensional culture from mouse embryonic stem cells, for example, is an exciting step forward (Koehler et al., 2013). Likewise, in the olfactory system, recognition of the regenerative potential of the neural crest-derived olfactory ensheathing glia has implications for the development of patient-based cell therapies for sensory and other disorders (Barraud et al., 2010).

More work remains to understand and reconcile the differences in otic and olfactory neurogenesis across various model systems. In addition, there is still much to learn about the timing and cellular interactions that allow system-wide organ development to proceed smoothly and precisely. A critical next step is to integrate our knowledge of signaling cascades with the complex, four-dimensional physical morphogenesis that occurs in the developing ear and nose during both development and regeneration. For example, our knowledge of mRNA expression and secreted protein gradients has grown rapidly, but these datasets often contain most of their information in just two dimensions. With the advent of increasingly powerful imaging technologies, it has become feasible to visualize protein expression, movement and interactions in vivo in the context of the cell-cell interactions that underlie organ morphogenesis and growth. Going forward, it will be exciting to map well-described molecular information onto this ‘developing’ spatiotemporal map of otic and olfactory morphogenesis more robustly and thus gain new, powerful insights into development and regeneration. Such four-dimensional mapping holds the promise of providing information useful for therapeutic purposes in the human ear and nose. Importantly, a greater general knowledge of neurogenesis will aid in promoting neuronal regeneration and repair both within and outside the otic and olfactory systems.

## References

[bib1] Abelló G., Khatri S., Giráldez F., Alsina B. (2007). Early regionalization of the otic placode and its regulation by the Notch signaling pathway. Mech. Dev..

[bib2] Abelló G., Khatri S., Radosevic M., Scotting P.J., Giráldez F., Alsina B. (2010). Independent regulation of Sox3 and Lmx1b by FGF and BMP signaling influences the neurogenic and non-neurogenic domains in the chick otic placode. Dev. Biol..

[bib3] Aburto M., Magariños M., Leon Y., Varela-Nieto I., Sanchez-Calderon H. (2012). AKT signaling mediates IGF-I survival actions on otic neural progenitors. PLoS One.

[bib4] Adam J., Myat A., Le Roux I., Eddison M., Henrique D., Ish-Horowicz D., Lewis J. (1998). Cell fate choices and the expression of Notch, Delta, and Serrate homologues in the chick inner ear: parallels with Drosophila sense organ development. Development.

[bib5] Adamska M., Herbrand H., Adamski M., Krüger M., Braun T., Bober E. (2001). FGFs control the patterning of the inner ear but are not able to induce the full ear program. Mech. Dev..

[bib6] Adamska M., Léger S., Brand M., Hadrys T., Braun T., Bober E. (2000). Inner ear and lateral line expression of a zebrafish Nkx5-1 gene and its downregulation in the ears of FGF8 mutant, ace. Mech. Dev..

[bib7] Adler H.J., Komeda M., Raphael Y. (1997). Further evidence for supporting cell conversion in the damaged avian basilar papilla. Int. J. Dev. Neurosci..

[bib8] Adler H.J., Raphael Y. (1996). New hair cells arise from supporting cell conversion in the acoustically damaged chick inner ear. Neurosci. Lett..

[bib9] Ahmed M., Wong E., Sun J., Xu J., Wang F., Xu P. (2012). Eya1–Six1 interaction is sufficient to induce hair cell fate in the cochlea by activating Atoh1 expression in cooperation with Sox2. Dev. Cell.

[bib10] Ahrens K., Schlosser G. (2005). Tissues and signals involved in the induction of placodal Six1 expression in *Xenopus laevis*. Dev. Biol..

[bib11] Ahuja G., Ivandic I., Saltürk M., Oka Y., Nadler W., Korsching S. (2013). Zebrafish crypt neurons project to a single, identified mediodorsal glomerulus. Sci. Rep..

[bib12] Allen-Sharpley M., Tjia M., Cramer K. (2013). Differential roles for EphA and EphB signaling in segregation and patterning of central vestibulocochlear nerve projections. PLoS One.

[bib13] Alsina B., Abelló G., Ulloa E., Henrique D., Pujades C., Giraldez F. (2004). FGF signaling is required for determination of otic neuroblasts in the chick embryo. Dev. Biol..

[bib14] Alsina B., Giraldez F., Pujades C. (2009). Patterning and cell fate in ear development. Int. J. Dev. Biol..

[bib15] Alsina B., Giraldez F., Varela-Nieto I. (2003). Growth factors and early development of otic neurons: interactions between intrinsic and extrinsic signals. Curr. Top. Dev. Biol..

[bib16] Alvarado D.M., Hawkins R.D., Bashiardes S., Veile R.A., Ku Y.C., Powder K.E., Spriggs M.K., Speck J.D., Warchol M.E., Lovett M. (2011). An RNA interference-based screen of transcription factor genes identifies pathways necessary for sensory regeneration in the avian inner ear. J. Neurosci..

[bib17] Andermann P., Ungos J., Raible D.W. (2002). Neurogenin1 defines zebrafish cranial sensory ganglia precursors. Dev. Biol..

[bib18] Asan E., Drenckhahn D. (2005). Immunocytochemical characterization of two types of microvillar cells in rodent olfactory epithelium. Histochem. Cell Biol..

[bib19] Avallone B., Porritiello M., Esposito D., Mutone R., Balsamo G., Marmo F. (2003). Evidence for hair cell regeneration in the crista ampullaris of the lizard *Podarcis sicula*. Hear. Res..

[bib20] Baird R.A., Burton M.D., Lysakowski A., Fashena D.S., Naeger R.A. (2000). Hair cell recovery in mitotically blocked cultures of the bullfrog saccule. Proc. Natl. Acad. Sci. USA.

[bib21] Baird R.A., Steyger P.S., Schuff N.R. (1996). Mitotic and nonmitotic hair cell regeneration in the bullfrog vestibular otolith organs. Ann. N. Y. Acad. Sci..

[bib22] Balak K.J., Corwin J.T., Jones J.E. (1990). Regenerated hair cells can originate from supporting cell progeny: evidence from phototoxicity and laser ablation experiments in the lateral line system. J. Neurosci..

[bib23] Bang P., Sewell W., Malicki J. (2001). Morphology and cell type heterogeneities of the inner ear epithelia in adult and juvenile zebrafish (*Danio rerio*). J. Comp. Neurol..

[bib24] Barraud P., Seferiadis A.A., Tyson L.D., Zwart M.F., Szabo-Rogers H.L., Ruhrberg C., Liu K.J., Baker C.V. (2010). Neural crest origin of olfactory ensheathing glia. Proc. Natl. Acad. Sci. USA.

[bib25] Batts S.A., Shoemaker C.R., Raphael Y. (2009). Notch signaling and Hes labeling in the normal and drug-damaged organ of Corti. Hear. Res..

[bib26] Bazáes A., Olivares J., Schmachtenberg O. (2013). Properties, projections, and tuning of teleost olfactory receptor neurons. J. Chem. Ecol..

[bib27] Beites C., Kawauchi S., Crocker C., Calof A. (2005). Identification and molecular regulation of neural stem cells in the olfactory epithelium. Exp. Cell Res..

[bib28] Bell D., Streit A., Gorospe I., Varela-Nieto I., Alsina B., Giraldez F. (2008). Spatial and temporal segregation of auditory and vestibular neurons in the otic placode. Dev. Biol..

[bib29] Bermingham N.A., Hassan B.A., Price S.D., Vollrath M.A., Ben-Arie N., Eatock R.A., Bellen H.J., Lysakowski A., Zoghbi H.Y. (1999). Math1: an essential gene for the generation of inner ear hair cells. Science.

[bib30] Bermingham-McDonogh O., Reh T.A. (2011). Regulated reprogramming in the regeneration of sensory receptor cells. Neuron.

[bib31] Bhasin N., Maynard T., Gallagher P., LaMantia A. (2003). Mesenchymal/epithelial regulation of retinoic acid signaling in the olfactory placode. Dev. Biol..

[bib32] Boddy S.L., Chen W., Romero-Guevara R., Kottam L., Bellantuono I., Rivolta M.N. (2012). Inner ear progenitor cells can be generated in vitro from human bone marrow mesenchymal stem cells. Regen. Med..

[bib33] Bohne B.A., Harding G.W. (1992). Neural regeneration in the noise-damaged chinchilla cochlea. Laryngoscope.

[bib34] Bok J., Bronner-Fraser M., Wu D.K. (2005). Role of the hindbrain in dorsoventral but not anteroposterior axial specification of the inner ear. Development.

[bib35] Bok J., Raft S., Kong K.A., Koo S.K., Dräger U.C., Wu D.K. (2011). Transient retinoic acid signaling confers anterior–posterior polarity to the inner ear. Proc. Natl. Acad. Sci. USA.

[bib36] Bok J., Zenczak C., Hwang C., Wu D. (2013). Auditory ganglion source of Sonic hedgehog regulates timing of cell cycle exit and differentiation of mammalian cochlear hair cells. Proc. Natl. Acad. Sci. USA.

[bib37] Bozza T., Vassalli A., Fuss S., Zhang J.J., Weiland B., Pacifico R., Feinstein P., Mombaerts P. (2009). Mapping of class I and class II odorant receptors to glomerular domains by two distinct types of olfactory sensory neurons in the mouse. Neuron.

[bib38] Brooker R., Hozumi K., Lewis J. (2006). Notch ligands with contrasting functions: Jagged1 and Delta1 in the mouse inner ear. Development.

[bib39] Brugeaud, A., Tong, M., Luo, L. & Edge, A.S. (2013). Inhibition of repulsive guidance molecule, RGMa, increases afferent synapse formation with auditory hair cells. Dev. Neurobiol. 10.1002/dneu.22136 PMC5540376 24123853

[bib40] Buck L., Axel R. (1991). A novel multigene family may encode odorant receptors: a molecular basis for odor recognition. Cell.

[bib41] Buck L.B. (2000). The molecular architecture of odor and pheromone sensing in mammals. Cell.

[bib42] Bulankina A., Moser T. (2012). Neural circuit development in the mammalian cochlea. Physiology (Bethesda).

[bib43] Burns J., Collado M., Oliver E., Corwin J. (2013). Specializations of intercellular junctions are associated with the presence and absence of hair cell regeneration in ears from six vertebrate classes. J. Comp. Neurol..

[bib44] Burns J.C., Christophel J.J., Collado M.S., Magnus C., Carfrae M., Corwin J.T. (2008). Reinforcement of cell junctions correlates with the absence of hair cell regeneration in mammals and its occurrence in birds. J. Comp. Neurol..

[bib45] Burns J.C., Corwin J.T. (2013). A historical to present-day account of efforts to answer the question: “what puts the brakes on mammalian hair cell regeneration?”. Hear. Res..

[bib46] Burns J.C., Cox B.C., Thiede B.R., Zuo J., Corwin J.T. (2012). In vivo proliferative regeneration of balance hair cells in newborn mice. J. Neurosci..

[bib47] Cafaro J., Lee G.S., Stone J.S. (2007). Atoh1 expression defines activated progenitors and differentiating hair cells during avian hair cell regeneration. Dev. Dyn..

[bib48] Cai T., Seymour M., Zhang H., Pereira F., Groves A. (2013). Conditional deletion of Atoh1 reveals distinct critical periods for survival and function of hair cells in the organ of Corti. J. Neurosci..

[bib49] Calof A., Bonnin A., Crocker C., Kawauchi S., Murray R., Shou J., Wu H. (2002). Progenitor cells of the olfactory receptor neuron lineage. Micros. Res. Tech..

[bib50] Camarero G., Avendano C., Fernandez-Moreno C., Villar A., Contreras J., de Pablo F., Pichel J., Varela-Nieto I. (2001). Delayed inner ear maturation and neuronal loss in postnatal Igf-1-deficient mice. J. Neurosci..

[bib51] Camarero G., Leon Y., Gorospe I., De Pablo F., Alsina B., Giraldez F., Varela-Nieto I. (2003). Insulin-like growth factor 1 is required for survival of transit-amplifying neuroblasts and differentiation of otic neurons. Dev. Biol..

[bib52] Cariboni A., Maggi R., Parnavelas J. (2007). From nose to fertility: the long migratory journey of gonadotropin-releasing hormone neurons. Trends Neurosci..

[bib53] Carney P., Silver J. (1983). Studies on cell migration and axon guidance in the developing distal auditory system of the mouse. J. Comp. Neurol..

[bib54] Carney T., Dutton K., Greenhill E., Delfino-Machín M., Dufourcq P., Blader P., Kelsh R. (2006). A direct role for Sox10 in specification of neural crest-derived sensory neurons. Development.

[bib55] Cau E., Casarosa S., Guillemot F. (2002). Mash1 and Ngn1 control distinct steps of determination and differentiation in the olfactory sensory neuron lineage. Development.

[bib56] Cau E., Gradwohl G., Casarosa S., Kageyama R., Guillemot F. (2000). Hes genes regulate sequential stages of neurogenesis in the olfactory epithelium. Development.

[bib57] Cau E., Gradwohl G., Fode C., Guillemot F. (1997). Mash1 activates a cascade of bHLH regulators in olfactory neuron progenitors. Development.

[bib58] Cediel R., Riquelme R., Contreras J., Díaz A., Varela-Nieto I. (2006). Sensorineural hearing loss in insulin-like growth factor I-null mice: a new model of human deafness. Eur. J. Neurosci..

[bib59] Chen P., Johnson J., Zoghbi H., Segil N. (2002). The role of Math1 in inner ear development: uncoupling the establishment of the sensory primordium from hair cell fate determination. Development.

[bib60] Chen W., Jongkamonwiwat N., Abbas L., Eshtan S., Johnson S., Kuhn S., Milo M., Thurlow J., Andrews P., Marcotti W., Moore H., Rivolta M. (2012). Restoration of auditory evoked responses by human ES-cell-derived otic progenitors. Nature.

[bib61] Chess A., Simon I., Cedar H., Axel R. (1994). Allelic inactivation regulates olfactory receptor gene expression. Cell.

[bib62] Chiappe M., Kozlov A., Hudspeth A. (2007). The structural and functional differentiation of hair cells in a lizard׳s basilar papilla suggests an operational principle of amniote cochleas. J. Neurosci..

[bib63] Chonko K., Jahan I., Stone J., Wright M., Fujiyama T., Hoshino M., Fritzsch B., Maricich S. (2013). Atoh1 directs hair cell differentiation and survival in the late embryonic mouse inner ear. Dev. Biol..

[bib64] Cole L., Le Roux I., Nunes F., Laufer E., Lewis J., Wu D. (2000). Sensory organ generation in the chicken inner ear: contributions of bone morphogenetic protein 4, serrate1, and lunatic fringe. J. Comp. Neurol..

[bib65] Coleman B., Fallon J.B., Pettingill L.N., de Silva M.G., Shepherd R.K. (2007). Auditory hair cell explant co-cultures promote the differentiation of stem cells into bipolar neurons. Exp. Cell Res..

[bib66] Collado M.S., Thiede B.R., Baker W., Askew C., Igbani L.M., Corwin J.T. (2011). The postnatal accumulation of junctional E-cadherin is inversely correlated with the capacity for supporting cells to convert directly into sensory hair cells in mammalian balance organs. J. Neurosci..

[bib67] Corey D.P. (2009). Cell biology of mechanotransduction in inner-ear hair cells. F1000 Biol. Rep..

[bib68] Corrales C.E., Pan L., Li H., Liberman M.C., Heller S., Edge A.S. (2006). Engraftment and differentiation of embryonic stem cell-derived neural progenitor cells in the cochlear nerve trunk: growth of processes into the organ of Corti. J. Neurobiol..

[bib69] Correia M., Lang D. (1990). An electrophysiological comparison of solitary type I and type II vestibular hair cells. Neurosci. Lett..

[bib70] Corwin J.T. (1981). Postembryonic production and aging in inner ear hair cells in sharks. J. Comp. Neurol..

[bib71] Corwin J.T. (1983). Postembryonic growth of the macula neglecta auditory detector in the ray, Raja clavata: continual increases in hair cell number, neural convergence, and physiological sensitivity. J. Comp. Neurol.

[bib72] Corwin J.T., Cotanche D.A. (1988). Regeneration of sensory hair cells after acoustic trauma. Science.

[bib73] Cotanche D.A. (1987). Regeneration of hair cell stereociliary bundles in the chick cochlea following severe acoustic trauma. Hear. Res..

[bib74] Couly G., Le Douarin N. (1985). Mapping of the early neural primordium in quail-chick chimeras. I. Developmental relationships between placodes, facial ectoderm, and prosencephalon. Dev. Biol..

[bib75] Croucher S., Tickle C. (1989). Characterization of epithelial domains in the nasal passages of chick embryos: spatial and temporal mapping of a range of extracellular matrix and cell surface molecules during development of the nasal placode. Development.

[bib76] Cruz R.M., Lambert P.R., Rubel E.W. (1987). Light microscopic evidence of hair cell regeneration after gentamicin toxicity in chick cochlea. Arch. Otolaryngol. Head Neck Surg..

[bib77] D’Amico-Martel A., Noden D.M. (1983). Contributions of placodal and neural crest cells to avian cranial peripheral ganglia. Am. J. Anat..

[bib78] Dallos P. (2008). Cochlear amplification, outer hair cells and prestin. Curr. Opin. Neurobiol..

[bib79] Dallos P., Zheng J., Cheatham M. (2006). Prestin and the cochlear amplifier. J. Physiol..

[bib80] Daudet N., Ariza-McNaughton L., Lewis J. (2007). Notch signaling is needed to maintain, but not to initiate, the formation of prosensory patches in the chick inner ear. Development.

[bib81] Daudet N., Lewis J. (2005). Two contrasting roles for Notch activity in chick inner ear development: specification of prosensory patches and lateral inhibition of hair-cell differentiation. Development.

[bib82] De Carlos J., López-Mascaraque L., Valverde F. (1995). The telencephalic vesicles are innervated by olfactory placode-derived cells: a possible mechanism to induce neocortical development. Neuroscience.

[bib83] DeHamer M., Guevara J., Hannon K., Olwin B., Calof A. (1994). Genesis of olfactory receptor neurons in vitro: regulation of progenitor cell divisions by fibroblast growth factors. Neuron.

[bib84] DeMaria S., Ngai J. (2010). The cell biology of smell. J. Cell Biol..

[bib85] Deol M. (1981). The inner ear in Bronx waltzer mice. Acta Oto-Laryngol..

[bib86] Donner A., Episkopou V., Maas R. (2007). Sox2 and Pou2f1 interact to control lens and olfactory placode development. Dev. Biol..

[bib87] Doty R.L. (2012). Olfactory dysfunction in Parkinson disease. Nat. Rev. Neurol..

[bib88] Doty R.L., Shaman P., Applebaum S.L., Giberson R., Siksorski L., Rosenberg L. (1984). Smell identification ability: changes with age. Science.

[bib89] Drapkin P., Silverman A. (1999). Development of the chick olfactory nerve. Dev. Dyn..

[bib90] Driver E., Pryor S., Hill P., Turner J., Rüther U., Biesecker L., Griffith A., Kelley M. (2008). Hedgehog signaling regulates sensory cell formation and auditory function in mice and humans. J. Neurosci..

[bib91] Driver E., Sillers L., Coate T., Rose M., Kelley M. (2013). The Atoh1-lineage gives rise to hair cells and supporting cells within the mammalian cochlea. Dev. Biol..

[bib92] Duggan C., DeMaria S., Baudhuin A., Stafford D., Ngai J. (2008). Foxg1 is required for development of the vertebrate olfactory system. J. Neurosci..

[bib93] Duncan J., Fritzsch B. (2013). Continued expression of GATA3 is necessary for cochlear neurosensory development. PLoS One.

[bib94] Duran Alonso M.B., Feijoo-Redondo A., Conde de Felipe M., Carnicero E., Garcia A.S., Garcia-Sancho J., Rivolta M.N., Giraldez F., Schimmang T. (2012). Generation of inner ear sensory cells from bone marrow-derived human mesenchymal stem cells. Regen. Med..

[bib95] Dutton K., Abbas L., Spencer J., Brannon C., Mowbray C., Nikaido M., Kelsh R.N., Whitfield T.T. (2009). A zebrafish model for Waardenburg syndrome type IV reveals diverse roles for Sox10 in the otic vesicle. Dis. Model Mech..

[bib96] Eatock R., Rüsch A., Lysakowski A., Saeki M. (1998). Hair cells in mammalian utricles. Otolaryngol. Head Neck Surg..

[bib97] Elsaesser R., Montani G., Tirindelli R., Paysan J. (2005). Phosphatidyl-inositide signaling proteins in a novel class of sensory cells in the mammalian olfactory epithelium. Eur. J. Neurosci..

[bib98] Elsaesser R., Paysan J. (2007). The sense of smell, its signaling pathways, and the dichotomy of cilia and microvilli in olfactory sensory cells. BMC Neurosci..

[bib99] Fantetti K., Fekete D. (2012). Members of the BMP, Shh, and FGF morphogen families promote chicken statoacoustic ganglion neurite outgrowth and neuron survival in vitro. Dev. Neurobiol..

[bib100] Fariñas I., Jones K., Tessarollo L., Vigers A., Huang E., Kirstein M., de Caprona D., Coppola V., Backus C., Reichardt L., Fritzsch B. (2001). Spatial shaping of cochlear innervation by temporally regulated neurotrophin expression. J. Neurosci..

[bib101] Fernandez C., Goldberg J.M., Baird R.A. (1990). The vestibular nerve of the chinchilla. III. Peripheral innervation patterns in the utricular macula. J. Neurophysiol..

[bib102] Fischer F. (1994). Quantitative TEM analysis of the barn owl basilar papilla. Hear. Res..

[bib103] Fletcher R.B., Prasol M.S., Estrada J., Baudhuin A., Vranizan K., Choi Y.G., Ngai J. (2011). p63 regulates olfactory stem cell self-renewal and differentiation. Neuron.

[bib104] Forge A. (1985). Outer hair cell loss and supporting cell expansion following chronic gentamicin treatment. Hear. Res..

[bib105] Forge A., Li L., Corwin J.T., Nevill G. (1993). Ultrastructural evidence for hair cell regeneration in the mammalian inner ear. Science.

[bib106] Forge A., Li L., Nevill G. (1998). Hair cell recovery in the vestibular sensory epithelia of mature guinea pigs. J. Comp. Neurol..

[bib107] Fornaro M., Geuna S., Fasolo A., Giacobini-Robecchi M. (2003). HuC/D confocal imaging points to olfactory migratory cells as the first cell population that expresses a post-mitotic neuronal phenotype in the chick embryo. Neuroscience.

[bib108] Forni P.E., Taylor-Burds C., Melvin V.S., Williams T., Wray S. (2011). Neural crest and ectodermal cells intermix in the nasal placode to give rise to GnRH-1 neurons, sensory neurons, and olfactory ensheathing cells. J. Neurosci..

[bib109] Freyer L., Aggarwal V., Morrow B.E. (2011). Dual embryonic origin of the mammalian otic vesicle forming the inner ear. Development.

[bib110] Fritzsch B., Beisel K., Hansen L. (2006). The molecular basis of neurosensory cell formation in ear development: a blueprint for hair cell and sensory neuron regeneration?. Bioessays.

[bib111] Fritzsch B., Eberl D., Beisel K. (2010). The role of bHLH genes in ear development and evolution: revisiting a 10-year-old hypothesis. Cell. Mol. Life Sci..

[bib112] Fritzsch B., Pauley S., Beisel K. (2006). Cells, molecules and morphogenesis: the making of the vertebrate ear. Brain Res..

[bib113] Gajewski M., Elmasri H., Girschick M., Sieger D., Winkler C. (2006). Comparative analysis of her genes during fish somitogenesis suggests a mouse/chick-like mode of oscillation in medaka. Dev. Genes Evol..

[bib114] Gayoso J., Castro A., Anadon R., Manso M. (2012). Crypt cells of the zebrafish *Danio rerio* mainly project to the dorsomedial glomerular field of the olfactory bulb. Chem. Senses.

[bib115] Girod D.A., Duckert L.G., Rubel E.W. (1989). Possible precursors of regenerated hair cells in the avian cochlea following acoustic trauma. Hear. Res..

[bib116] Gokoffski K., Kawauchi S., Wu H., Santos R., Hollenbeck P., Lander A., Calof A., Menini A. (2010). Feedback regulation of neurogenesis in the mammalian olfactory epithelium: new insights from genetics and systems biology. The Neurobiology of Olfaction.

[bib117] Gokoffski K., Wu H., Beites C., Kim J., Kim E., Matzuk M., Johnson J., Lander A., Calof A. (2011). Activin and GDF11 collaborate in feedback control of neuroepithelial stem cell proliferation and fate. Development.

[bib118] Goldberg J.M., Lysakowski A., Fernandez C. (1990). Morphophysiological and ultrastructural studies in the mammalian cristae ampullares. Hear. Res..

[bib119] Gordon M., Mumm J., Davis R., Holcomb J., Calof A. (1995). Dynamics of MASH1 expression in vitro and in vivo suggest a non-stem cell site of MASH1 action in the olfactory receptor neuron lineage. Mol. Cell. Neurosci..

[bib120] Goto F., Straka H., Dieringer N. (2002). Gradual and reversible central vestibular reorganization in frog after selective labyrinthine nerve branch lesions. Exp. Brain Res..

[bib121] Graham A., Blentic A., Duque S., Begbie J. (2007). Delamination of cells from neurogenic placodes does not involve an epithelial-to-mesenchymal transition. Development.

[bib122] Graziadei G.A., Graziadei P.P. (1979). Neurogenesis and neuron regeneration in the olfactory system of mammals. II. Degeneration and reconstitution of the olfactory sensory neurons after axotomy. J. Neurocytol..

[bib123] Groves A.K., LaBonne C. (2014). Setting appropriate boundaries: fate, patterning and competence at the neural plate border. Dev. Biol..

[bib124] Gu C., Rodriguez E., Reimert D., Shu T., Fritzsch B., Richards L., Kolodkin A., Ginty D. (2003). Neuropilin-1 conveys semaphorin and VEGF signaling during neural and cardiovascular development. Dev. Cell.

[bib125] Guillemot F., Lo L.-C., Johnson J.E., Auerbach A., Anderson D.J., Joyner A.L. (1993). Mammalian achaete-scute homolog 1 is required for the early development of olfactory and autonomic neurons. Cell.

[bib126] Guo Z., Packard A., Krolewski R., Harris M., Manglapus G., Schwob J. (2010). Expression of pax6 and sox2 in adult olfactory epithelium. J. Comp. Neurol..

[bib127] Haddon C., Jiang Y.-J., Smithers L., Lewis J. (1998). Delta-Notch signalling and the patterning of sensory cell differentiation in the zebrafish ear: evidence from the mind bomb mutant. Development.

[bib128] Haddon C., Lewis J. (1996). Early ear development in the embryo of the zebrafish *Danio rerio*. J. Comp. Neurol..

[bib129] Haddon C., Mowbray C., Whitfield T., Jones D., Gschmeissner S., Lewis J. (1999). Hair cells without supporting cells: further studies in the ear of the zebrafish mind bomb mutant. J. Neurocytol..

[bib130] Haden M., Einarsson R., Yazejian B. (2013). Patch clamp recordings of hair cells isolated from zebrafish auditory and vestibular end organs. Neuroscience.

[bib131] Hammond K.L., van Eeden F.J., Whitfield T.T. (2010). Repression of Hedgehog signaling is required for the acquisition of dorsolateral cell fates in the zebrafish otic vesicle. Development.

[bib132] Hammond K.L., Whitfield T.T. (2011). Fgf and Hh signaling act on a symmetrical pre-pattern to specify anterior and posterior identity in the zebrafish otic placode and vesicle. Development.

[bib133] Hans S., Irmscher A., Brand M. (2013). Zebrafish Foxi1 provides a neuronal ground state during inner ear induction preceding the Dlx3b/4b-regulated sensory lineage. Development.

[bib134] Hansen A., Finger T. (2008). Is TrpM5 a reliable marker for chemosensory cells? Multiple types of microvillous cells in the main olfactory epithelium of mice. BMC Neurosci..

[bib135] Hansen A., Zeiske E. (1998). The peripheral olfactory organ of the zebrafish, *Danio rerio*: an ultrastructural study. Chem. Senses.

[bib136] Hansen A., Zielinski B. (2005). Diversity in the olfactory epithelium of bony fishes: development, lamellar arrangement, sensory neuron cell types and transduction components. J. Neurocytol..

[bib137] Hashino E., Tanaka Y., Salvi R.J., Sokabe M. (1992). Hair cell regeneration in the adult budgerigar after kanamycin ototoxicity. Hear. Res..

[bib138] Hawkins R.D., Bashiardes S., Powder K.E., Sajan S.A., Bhonagiri V., Alvarado D.M., Speck J., Warchol M.E., Lovett M. (2007). Large scale gene expression profiles of regenerating inner ear sensory epithelia. PLoS One.

[bib139] Hegg C., Jia C., Chick W., Restrepo D., Hansen A. (2010). Microvillous cells expressing IP3 receptor type 3 in the olfactory epithelium of mice. Eur. J. Neurosci..

[bib140] Hemond S.G., Morest D.K. (1991). Ganglion formation from the otic placode and the otic crest in the chick embryo: mitosis, migration, and the basal lamina. Anat. Embryol..

[bib141] Hennig A.K., Cotanche D.A. (1998). Regeneration of cochlear efferent nerve terminals after gentamycin damage. J. Neurosci..

[bib142] Hinds J., Hinds P., McNelly N. (1984). An autoradiographic study of the mouse olfactory epithelium: evidence for long-lived receptors. Anat. Rec..

[bib143] Hirota J., Mombaerts P. (2004). The LIM-homeodomain protein Lhx2 is required for complete development of mouse olfactory sensory neurons. Proc. Natl. Acad. Sci. USA.

[bib144] Hirota J., Omura M., Mombaerts P. (2007). Differential impact of Lhx2 deficiency on expression of class I and class II odorant receptor genes in mouse. Mol. Cell. Neurosci..

[bib145] Holley M., Rhodes C., Kneebone A., Herde M.K., Fleming M., Steel K.P. (2010). Emx2 and early hair cell development in the mouse inner ear. Dev. Biol..

[bib146] Hoppe R., Frank H., Breer H., Strotmann J. (2003). The clustered olfactory receptor gene family 262: genomic organization, promotor elements, and interacting transcription factors. Genome Res..

[bib147] Hossain W., Zhou X., Rutledge A., Baier C., Morest D. (1996). Basic fibroblast growth factor affects neuronal migration and differentiation in normotypic cell cultures from the cochleovestibular ganglion of the chick embryo. Exp. Neurol..

[bib148] Hsu P., Yu F., Féron F., Pickles J., Sneesby K., Mackay-Sim A. (2001). Basic fibroblast growth factor and fibroblast growth factor receptors in adult olfactory epithelium. Brain Res..

[bib149] Hu Z., Andang M., Ni D., Ulfendahl M. (2005). Neural cograft stimulates the survival and differentiation of embryonic stem cells in the adult mammalian auditory system. Brain Res..

[bib150] Hu Z., Luo X., Zhang L., Lu F., Dong F., Monsell E., Jiang H. (2012). Generation of human inner ear prosensory-like cells via epithelial-to-mesenchymal transition. Regen. Med..

[bib151] Huang E., Liu W., Fritzsch B., Bianchi L., Reichardt L., Xiang M. (2001). Brn3a is a transcriptional regulator of soma size, target field innervation and axon pathfinding of inner ear sensory neurons. Development.

[bib152] Huh S., Jones J., Warchol M., Ornitz D. (2012). Differentiation of the lateral compartment of the cochlea requires a temporally restricted FGF20 signal. PLoS Biol..

[bib153] Irvine D.R., Brown M., Kamke M.R., Rubel E.W. (2009). Effects of restricted basilar papillar lesions and hair cell regeneration on auditory forebrain frequency organization in adult European starlings. J. Neurosci..

[bib154] Iwai N., Zhou Z., Roop D.R., Behringer R.R. (2008). Horizontal basal cells are multipotent progenitors in normal and injured adult olfactory epithelium. Stem Cells.

[bib155] Izumikawa M., Minoda R., Kawamoto K., Abrashkin K.A., Swiderski D.L., Dolan D.F., Brough D.E., Raphael Y. (2005). Auditory hair cell replacement and hearing improvement by Atoh1 gene therapy in deaf mammals. Nat. Med.

[bib156] Jacques, B., Montgomery, W.H. 4th, Uribe, P., Yatteau, A., Asuncion, J., Resendiz, G., Matsui, J., Dabdoub, A., The role of Wnt/ß-catenin signaling in proliferation and regeneration of the developing basilar papilla and lateral line. Dev. Neurobiol 10.1002/dneu.22134, in press. 10.1002/dneu.22134 24115534

[bib157] Jeon S., Fujioka M., Kim S., Edge A. (2011). Notch signaling alters sensory or neuronal cell fate specification of inner ear stem cells. J. Neurosci..

[bib158] Jia C., Hayoz S., Hutch C.R., Iqbal T.R., Pooley A.E., Hegg C.C. (2013). An IP3R3- and NPY-expressing microvillous cell mediates tissue homeostasis and regeneration in the mouse olfactory epithelium. PLoS One.

[bib159] Johnsson L., Hawkins J.J. (1972). Strial atrophy in clinical and experimental deafness. Laryngoscope.

[bib160] Jones J., Warchol M. (2009). Expression of the Gata3 transcription factor in the acoustic ganglion of the developing avian inner ear. J. Comp. Neurol..

[bib161] Jørgensen J.M., Mathiesen C. (1988). The avian inner ear. Continuous production of hair cells in vestibular sensory organs, but not in the auditory papilla. Naturwissenschaften.

[bib162] Kaiser C.L., Kamien A.J., Shah P.A., Chapman B.J., Cotanche D.A. (2009). 5-Ethynyl-2′-deoxyuridine labeling detects proliferating cells in the regenerating avian cochlea. Laryngoscope.

[bib163] Kamaid A., Neves J., Giráldez F. (2010). Id gene regulation and function in the prosensory domains of the chicken inner ear: a link between Bmp signaling and Atoh1. J. Neurosci..

[bib164] Karis A., Pata I., van Doorninck J., Grosveld F., de Zeeuw C., de Caprona D., Fritzsch B. (2001). Transcription factor GATA-3 alters pathway selection of olivocochlear neurons and affects morphogenesis of the ear. J. Comp. Neurol..

[bib165] Katayama K., Imai F., Suto F., Yoshida Y. (2013). Deletion of Sema3a or plexinA1/plexinA3 causes defects in sensory afferent projections of statoacoustic ganglion neurons. PLoS One.

[bib166] Katoh H., Shibata S., Fukuda K., Sato M., Satoh E., Nagoshi N., Minematsu T., Matsuzaki Y., Akazawa C., Toyama Y., Nakamura M., Okano H. (2011). The dual origin of the peripheral olfactory system: placode and neural crest. Mol. Brain.

[bib167] Kawai T., Oka Y., Eisthen H. (2009). The role of the terminal nerve and GnRH in olfactory system neuromodulation. Zool. Sci..

[bib168] Kawaja M., Boyd J., Smithson L., Jahed A., Doucette R. (2009). Technical strategies to isolate olfactory ensheathing cells for intraspinal implantation. J. Neurotrauma.

[bib169] Kawamoto K., Ishimoto S., Minoda R., Brough D.E., Raphael Y. (2003). Math1 gene transfer generates new cochlear hair cells in mature guinea pigs in vivo. J. Neurosci..

[bib170] Kawauchi S., Beites C., Crocker C., Wu H., Bonnin A., Murray R., Calof A. (2004). Molecular signals regulating proliferation of stem and progenitor cells in mouse olfactory epithelium. Dev. Neurosci..

[bib171] Kawauchi S., Santos R., Kim J., Hollenbeck P., Murray R., Calof A. (2009). The role of foxg1 in the development of neural stem cells of the olfactory epithelium. Ann. N. Y. Acad. Sci..

[bib172] Kawauchi S., Shou J., Santos R., Hébert J., McConnell S.I.M., Calof A. (2005). Fgf8 expression defines a morphogenetic center required for olfactory neurogenesis and nasal cavity development in the mouse. Development.

[bib173] Kazmierczak P., Müller U. (2012). Sensing sound: molecules that orchestrate mechanotransduction by hair cells. Trends Neurosci..

[bib174] Kelley M. (2006). Hair cell development: commitment through differentiation. Brain Res..

[bib175] Kelley M. (2006). Regulation of cell fate in the sensory epithelia of the inner ear. Nat. Rev. Neurosci.

[bib176] Kelley M., Driver E., Puligilla C. (2009). Regulation of cell fate and patterning in the developing mammalian cochlea. Curr. Opin. Otolaryngol. Head Neck Surg..

[bib177] Kelley M.W., Talreja D.R., Corwin J.T. (1995). Replacement of hair cells after laser microbeam irradiation in cultured organs of Corti from embryonic and neonatal mice. J. Neurosci..

[bib178] Kelly M., Chang Q., Pan A., Lin X., Chen P. (2012). Atoh1 directs the formation of sensory mosaics and induces cell proliferation in the postnatal mammalian cochlea in vivo. J. Neurosci..

[bib179] Khan M., Vaes E., Mombaerts P. (2011). Regulation of the probability of mouse odorant receptor gene choice. Cell.

[bib180] Kiernan A., Pelling A., Leung K., Tang A., Bell D., Tease C., Lovell-Badge R., Steel K., Cheah K. (2005). Sox2 is required for sensory organ development in the mammalian inner ear. Nature.

[bib181] Kiernan A., Xu J., Gridley T. (2006). The Notch ligand JAG1 is required for sensory progenitor development in the mammalian inner ear. PLoS Genet.

[bib182] Kiernan A.E., Ahituv N., Fuchs H., Balling R., Avraham K.B., Steel K.P., Hrabé de Angelis M. (2001). The Notch ligand Jagged1 is required for inner ear sensory development. Proc. Natl. Acad. Sci. USA.

[bib183] Kim T.S., Nakagawa T., Kita T., Higashi T., Takebayashi S., Matsumoto M., Kojima K., Sakamoto T., Ito J. (2005). Neural connections between embryonic stem cell-derived neurons and vestibular hair cells in vitro. Brain Res..

[bib184] Kim W.Y., Fritzsch B., Serls A., Bakel L.A., Huang E.J., Reichardt L.F., Barth D.S., Lee J.E. (2001). NeuroD-null mice are deaf due to a severe loss of the inner ear sensory neurons during development. Development.

[bib185] Koehler K., Mikosz A., Molosh A., Patel D., Hashino E. (2013). Generation of inner ear sensory epithelia from pluripotent stem cells in 3D culture. Nature.

[bib186] Kolterud A., Alenius M., Carlsson L., Bohm S. (2004). The Lim homeobox gene Lhx2 is required for olfactory sensory neuron identity. Development.

[bib187] Koo S., Hill J., Hwang C., Lin Z., Millen K., Wu D. (2009). Lmx1a maintains proper neurogenic, sensory, and non-sensory domains in the mammalian inner ear. Dev. Biol..

[bib188] Köppl C. (2011). Birds—same thing, but different? Convergent evolution in the avian and mammalian auditory systems provides informative comparative models. Hear. Res..

[bib189] Kujawa S.G., Liberman M.C. (2009). Adding insult to injury: cochlear nerve degeneration after “temporary” noise-induced hearing loss. J. Neurosci..

[bib190] Kuntz A.L., Oesterle E.C. (1998). Transforming growth factor-alpha with insulin induces proliferation in rat utricular extrasensory epithelia. Otolaryngol. Head Neck Surg..

[bib191] LaMantia A., Bhasin N., Rhodes K., Heemskerk J. (2000). Mesenchymal/epithelial induction mediates olfactory pathway formation. Neuron.

[bib192] Lanford P., Lan Y., Jiang R., Lindsell C., Weinmaster G., Gridley T., Kelley M. (1999). Notch signalling pathway mediates hair cell development in mammalian cochlea. Nat. Genet..

[bib193] Lawner B.E., Harding G.W., Bohne B.A. (1997). Time course of nerve-fiber regeneration in the noise-damaged mammalian cochlea. Int. J. Dev. Neurosci..

[bib194] Lawoko-Kerali G., Rivolta M., Lawlor P., Cacciabue-Rivolta D., Langton-Hewer C., van Doorninck J., Holley M. (2004). GATA3 and NeuroD distinguish auditory and vestibular neurons during development of the mammalian inner ear. Mech. Dev..

[bib195] Léger S., Brand M. (2002). Fgf8 and Fgf3 are required for zebrafish ear placode induction, maintenance and inner ear patterning. Mech. Dev..

[bib196] Leung C.T., Coulombe P.A., Reed R.R. (2007). Contribution of olfactory neural stem cells to tissue maintenance and regeneration. Nat. Neurosci..

[bib197] Lewis A., Vasudevan H., O’Neill A., Soriano P., Bush J. (2013). The widely used Wnt1–Cre transgene causes developmental phenotypes by ectopic activation of Wnt signaling. Dev. Biol..

[bib198] Lewis R.M., Hume C.R., Stone J.S. (2012). Atoh1 expression and function during auditory hair cell regeneration in post-hatch chickens. Hear. Res..

[bib199] Li H., Roblin G., Liu H., Heller S. (2003). Generation of hair cells by stepwise differentiation of embryonic stem cells. Proc. Natl. Acad. Sci. USA.

[bib200] Li L., Forge A. (1997). Morphological evidence for supporting cell to hair cell conversion in the mammalian utricular macula. Int. J. Dev. Neurosci..

[bib201] Liang J., Wang D., Renaud G., Wolfsberg T., Wilson A., Burgess S. (2012). The stat3/socs3a pathway is a key regulator of hair cell regeneration in zebrafish. J. Neurosci..

[bib202] Lin H.W., Furman A.C., Kujawa S.G., Liberman M.C. (2011). Primary neural degeneration in the Guinea pig cochlea after reversible noise-induced threshold shift. J. Assoc. Res. Otolaryngol..

[bib203] Lin V., Golub J., Nguyen T., Hume C., Oesterle E., Stone J. (2011). Inhibition of Notch activity promotes nonmitotic regeneration of hair cells in the adult mouse utricles. J. Neurosci..

[bib204] Lin W., Ezekwe E., Zhao Z., Liman E., Restrepo D. (2008). TRPM5-expressing microvillous cells in the main olfactory epithelium. BMC Neurosci..

[bib205] Lin Z., Perez P., Sun Z., Liu J.J., Shin J.H., Hyrc K.L., Samways D., Egan T., Holley M.C., Bao J. (2012). Reprogramming of single-cell-derived mesenchymal stem cells into hair cell-like cells. Otol. Neurotol..

[bib206] Liu Z., Dearman J.A., Cox B.C., Walters B.J., Zhang L., Ayrault O., Zindy F., Gan L., Roussel M.F., Zuo J. (2012). Age-dependent in vivo conversion of mouse cochlear pillar and Deiters׳ cells to immature hair cells by Atoh1 ectopic expression. J. Neurosci..

[bib207] Liu Z., Owen T., Zhang L., Zuo J. (2010). Dynamic expression pattern of Sonic hedgehog in developing cochlear spiral ganglion neurons. Dev. Dyn..

[bib208] Lombarte A., Yan H.Y., Popper A.N., Chang J.S., Platt C. (1993). Damage and regeneration of hair cell ciliary bundles in a fish ear following treatment with gentamicin. Hear. Res..

[bib209] Lu C., Appler J., Houseman E., Goodrich L. (2011). Developmental profiling of spiral ganglion neurons reveals insights into auditory circuit assembly. J. Neurosci..

[bib210] Lysakowski A., Goldberg J. (1997). A regional ultrastructural analysis of the cellular and synaptic architecture in the chinchilla cristae ampullares. J. Comp. Neurol..

[bib211] Lysakowski A., Minor L., Fernández C., Goldberg J. (1995). Physiological identification of morphologically distinct afferent classes innervating the cristae ampullares of the squirrel monkey. J. Neurophysiol..

[bib212] Ma E.Y., Rubel E.W., Raible D.W. (2008). Notch signaling regulates the extent of hair cell regeneration in the zebrafish lateral line. J. Neurosci..

[bib213] Ma Q., Chen Z., del Barco Barrantes I., de la Pompa J., Anderson D. (1998). neurogenin1 is essential for the determination of neuronal precursors for proximal cranial sensory ganglia. Neuron.

[bib214] Mackay-Sim A., Kittel P. (1991). On the life span of olfactory receptor neurons. Eur. J. Neurosci..

[bib215] Maier E. (2009). Early Development of the Olfactory Placode and Early Rostrocaudal Patterning of the Caudal Neural Tube (Ph.D. thesis).

[bib216] Maier E., Gunhaga L. (2009). Dynamic expression of neurogenic markers in the developing chick olfactory epithelium. Dev. Dyn..

[bib217] Maier E., Nord H., von Hofsten J., Gunhaga L. (2011). A balance of BMP and notch activity regulates neurogenesis and olfactory nerve formation. PLoS One.

[bib218] Maier E., von Hofsten J., Nord H., Fernandes M., Paek H., Hébert J., Gunhaga L. (2010). Opposing Fgf and Bmp activities regulate the specification of olfactory sensory and respiratory epithelial cell fates. Development.

[bib219] Malnic B., Hirono J., Sato T., Buck L.B. (1999). Combinatorial receptor codes for odors. Cell.

[bib220] Manley G. (2011). Lizard auditory papillae: an evolutionary kaleidoscope. Hear. Res..

[bib221] Mansour S.L., Goddard J.M., Capecchi M.R. (1993). Mice homozygous for a targeted disruption of the proto-oncogene int-2 have developmental defects in the tail and inner ear. Development.

[bib222] Marsh R.R., Xu L.R., Moy J.P., Saunders J.C. (1990). Recovery of the basilar papilla following intense sound exposure in the chick. Hear. Res..

[bib223] Martinez-Monedero R., Corrales C.E., Cuajungco M.P., Heller S., Edge A.S. (2006). Reinnervation of hair cells by auditory neurons after selective removal of spiral ganglion neurons. J. Neurobiol..

[bib224] Martinez-Monedero R., Yi E., Oshima K., Glowatzki E., Edge A.S. (2008). Differentiation of inner ear stem cells to functional sensory neurons. Dev. Neurobiol..

[bib225] Matsumoto M., Nakagawa T., Kojima K., Sakamoto T., Fujiyama F., Ito J. (2008). Potential of embryonic stem cell-derived neurons for synapse formation with auditory hair cells. J. Neurosci. Res..

[bib226] McIntyre J.C., Bose S.C., Stromberg A.J., McClintock T.S. (2008). Emx2 stimulates odorant receptor gene expression. Chem. Senses.

[bib227] Mensinger A.F., Highstein S.M. (1999). Characteristics of regenerating horizontal semicircular canal afferent and efferent fibers in the toadfish, *Opsanus tau*. J. Comp. Neurol..

[bib228] Miller A., Treloar H., Greer C. (2010). Composition of the migratory mass during development of the olfactory nerve. J. Comp. Neurol..

[bib229] Millimaki B.B., Sweet E.M., Dhason M.S., Riley B.B. (2007). Zebrafish atoh1 genes: classic proneural activity in the inner ear and regulation by Fgf and Notch. Development.

[bib230] Miyasaka N., Wanner A., Li J., Mack-Bucher J., Genoud C., Yoshihara Y., Friedrich R. (2013). Functional development of the olfactory system in zebrafish. Mech. Dev..

[bib231] Mizutari K., Fujioka M., Hosoya M., Bramhall N., Okano H., Okano H., Edge A. (2013). Notch inhibition induces cochlear hair cell regeneration and recovery of hearing after acoustic trauma. Neuron.

[bib232] Moravec W., Peterson E. (2004). Differences between stereocilia numbers on type I and type II vestibular hair cells. J. Neurophysiol..

[bib233] Mori K., Takahashi Y.K., Igarashi K.M., Yamaguchi M. (2006). Maps of odorant molecular features in the mammalian olfactory bulb. Phys. Rev..

[bib234] Morsli H., Choo D., Ryan A., Johnson R., Wu D.K. (1998). Development of the mouse inner ear and origin of its sensory organs. J. Neurosci..

[bib235] Mowbray C., Hammerschmidt M., Whitfield T.T. (2001). Expression of BMP signalling pathway members in the developing zebrafish inner ear and lateral line. Mech. Dev..

[bib236] Murray R., Navi D., Fesenko J., Lander A., Calof A. (2003). Widespread defects in the primary olfactory pathway caused by loss of Mash1 function. J. Neurosci..

[bib237] Nakano Y., Jahan I., Bonde G., Sun X., Hildebrand M., Engelhardt J., Smith R., Cornell R., Fritzsch B., Bánfi B. (2012). A mutation in the Srrm4 gene causes alternative splicing defects and deafness in the Bronx waltzer mouse. PLoS Genet..

[bib238] Nakashima M., Toyono T., Akamine A., Joyner A. (1999). Expression of growth/differentiation factor 11, a new member of the BMP/TGFbeta superfamily during mouse embryogenesis. Mech. Dev..

[bib239] Nayagam B., Muniak M., Ryugo D. (2011). The spiral ganglion: connecting the peripheral and central auditory systems. Hear. Res..

[bib240] Neves J., Abelló G., Petrovic J., Giraldez F. (2013). Patterning and cell fate in the inner ear: a case for Notch in the chicken embryo. Dev. Growth Differ..

[bib241] Neves J., Uchikawa M., Bigas A., Giraldez F. (2012). The prosensory function of Sox2 in the chicken inner ear relies on the direct regulation of Atoh1. PLoS One.

[bib242] Newman A., Suarez C., Kuruvilla A., Honrubia V. (1989). Regeneration of the eighth cranial nerve. III. Central projections of the primary afferent fibers from individual vestibular receptors in the bullfrog. Laryngoscope.

[bib243] Niederreither K., Fraulob V., Garnier J., Chambon P., Dollé P. (2002). Differential expression of retinoic acid-synthesizing (RALDH) enzymes during fetal development and organ differentiation in the mouse. Mech. Dev..

[bib244] Nikaido M., Doi K., Shimizu T., Hibi M., Kikuchi Y., Yamasu K. (2007). Initial specification of the epibranchial placode in zebrafish embryos depends on the fibroblast growth factor signal. Dev. Dyn..

[bib245] Norlin E.M., Alenius M., Gussing F., Hagglund M., Vedin V., Bohm S. (2001). Evidence for gradients of gene expression correlating with zonal topography of the olfactory sensory map. Mol. Cell. Neurosci..

[bib246] Oesterle E.C., Cunningham D.E., Westrum L.E., Rubel E.W. (2003). Ultrastructural analysis of [^3^H]thymidine-labeled cells in the rat utricular macula. J. Comp. Neurol..

[bib247] Ofsie M.S., Cotanche D.A. (1996). Distribution of nerve fibers in the basilar papilla of normal and sound-damaged chick cochleae. J. Comp. Neurol..

[bib248] Ogata Y., Slepecky N.B., Takahashi M. (1999). Study of the gerbil utricular macula following treatment with gentamicin, by use of bromodeoxyuridine and calmodulin immunohistochemical labelling. Hear. Res..

[bib249] Ogura T., Szebenyi S., Krosnowski K., Sathyanesan A., Jackson J., Lin W. (2011). Cholinergic microvillous cells in the mouse main olfactory epithelium and effect of acetylcholine on olfactory sensory neurons and supporting cells. J. Neurophysiol..

[bib250] Oka Y., Saraiva L., Korsching S. (2012). Crypt neurons express a single V1R-related ora gene. Chem. Senses.

[bib251] Okano T., Nakagawa T., Endo T., Kim T.S., Kita T., Tamura T., Matsumoto M., Ohno T., Sakamoto T., Iguchi F., Ito J. (2005). Engraftment of embryonic stem cell-derived neurons into the cochlear modiolus. Neuroreport.

[bib252] Oshima K., Grimm C.M., Corrales C.E., Senn P., Martinez Monedero R., Geleoc G.S., Edge A., Holt J.R., Heller S. (2007). Differential distribution of stem cells in the auditory and vestibular organs of the inner ear. J. Assoc. Res. Otolaryngol..

[bib253] Ouji Y., Ishizaka S., Nakamura-Uchiyama F., Yoshikawa M. (2012). In vitro differentiation of mouse embryonic stem cells into inner ear hair cell-like cells using stromal cell conditioned medium. Cell Death Dis..

[bib254] Packard A., Schnittke N., Romano R.A., Sinha S., Schwob J.E. (2011). DeltaNp63 regulates stem cell dynamics in the mammalian olfactory epithelium. J. Neurosci..

[bib255] Pan N., Jahan I., Kersigo J., Duncan J.S., Kopecky B., Fritzsch B. (2012). A novel Atoh1 “self-terminating” mouse model reveals the necessity of proper Atoh1 level and duration for hair cell differentiation and viability. PLoS One.

[bib256] Paschaki M., Cammas L., Muta Y., Matsuoka Y., Mak S., Rataj-Baniowska M., Fraulob V., Dollé P., Ladher R. (2013). Retinoic acid regulates olfactory progenitor cell fate and differentiation. Neural Dev..

[bib257] Peluso C.E., Jang W., Drager U.C., Schwob J.E. (2012). Differential expression of components of the retinoic acid signaling pathway in the adult mouse olfactory epithelium. J. Comp. Neurol..

[bib258] Pirvola U., Spencer-Dene B., Xing-Qun L., Kettunen P., Thesleff I., Fritzsch B., Dickson C., Ylikoski J. (2000). FGF/FGFR-2(IIIb) signaling is essential for inner ear morphogenesis. J. Neurosci..

[bib259] Pirvola U., Ylikoski J., Trokovic R., Hebert J.M., McConnell S.K., Partanen J. (2002). FGFR1 is required for the development of the auditory sensory epithelium. Neuron.

[bib260] Popper A.N., Hoxter B. (1984). Growth of a fish ear: 1. Quantitative analysis of hair cell and ganglion cell proliferation. Hear. Res..

[bib261] Pujades C., Kamaid A., Alsina B., Giraldez F. (2006). BMP-signaling regulates the generation of hair-cells. Dev. Biol..

[bib262] Qasba P., Reed R.R. (1998). Tissue and zonal-specific expression of an olfactory receptor transgene. J. Neurosci..

[bib263] Radde-Gallwitz K., Pan L., Gan L., Lin X., Segil N., Chen P. (2004). Expression of Islet1 marks the sensory and neuronal lineages in the mammalian inner ear. J. Comp. Neurol..

[bib264] Radosevic M., Robert-Moreno A., Coolen M., Bally-Cuif L., Alsina B. (2011). Her9 represses neurogenic fate downstream of Tbx1 and retinoic acid signaling in the inner ear. Development.

[bib265] Raft S., Koundakjian E., Quinones H., Jayasena C., Goodrich L., Johnson J., Segil N., Groves A. (2007). Cross-regulation of Ngn1 and Math1 coordinates the production of neurons and sensory hair cells during inner ear development. Development.

[bib266] Raft S., Nowotschin S., Liao J., Morrow B.E. (2004). Suppression of neural fate and control of inner ear morphogenesis by Tbx1. Development.

[bib267] Raphael Y. (1992). Evidence for supporting cell mitosis in response to acoustic trauma in the avian inner ear. J. Neurocytol..

[bib268] Raphael Y., Altschuler R. (2003). Structure and innervation of the cochlea. Brain Res. Bull..

[bib269] Ressler K.J., Sullivan S.L., Buck L.B. (1993). A zonal organization of odorant receptor gene expression in the olfactory epithelium. Cell.

[bib270] Riccomagno M.M., Martinu L., Mulheisen M., Wu D.K., Epstein D.J. (2002). Specification of the mammalian cochlea is dependent on Sonic hedgehog. Genes Dev..

[bib271] Richardson G., de Monvel J., Petit C. (2011). How the genetics of deafness illuminates auditory physiology. Ann. Rev. Physiol..

[bib272] Riquelme R., Cediel R., Contreras J., la Rosa Lourdes R., Murillo-Cuesta S., Hernandez-Sanchez C., Zubeldia J., Cerdan S., Varela-Nieto I. (2010). A comparative study of age-related hearing loss in wild type and insulin-like growth factor I deficient mice. Front. Neuroanat..

[bib273] Roberson D.F., Weisleder P., Bohrer P.S., Rubel E.W. (1992). Ongoing production of sensory cells in the vestibular epithelium of the chick. Hear. Res..

[bib274] Roberson D.W., Alosi J.A., Cotanche D.A. (2004). Direct transdifferentiation gives rise to the earliest new hair cells in regenerating avian auditory epithelium. J. Neurosci. Res..

[bib275] Roberson D.W., Rubel E.W. (1994). Cell division in the gerbil cochlea after acoustic trauma. Am. J. Otol..

[bib276] Rodriguez I. (2013). Singular expression of olfactory receptor genes. Cell.

[bib277] Rowley J., Moran D., Jafek B. (1989). Peroxidase backfills suggest the mammalian olfactory epithelium contains a second morphologically distinct class of bipolar sensory neuron: the microvillar cell. Brain Res..

[bib278] Rubel E.W., Dew L.A., Roberson D.W. (1995). Mammalian vestibular hair cell regeneration. Science.

[bib279] Rubel E.W., Furrer S.A., Stone J.S. (2013). A brief history of hair cell regeneration research and speculations on the future. Hear. Res..

[bib280] Ryals B.M., Rubel E.W. (1988). Hair cell regeneration after acoustic trauma in adult Coturnix quail. Science.

[bib281] Ryals B.M., Westbrook E.W. (1994). TEM analysis of neural terminals on autoradiographically identified regenerated hair cells. Hear. Res..

[bib282] Ryals B.M., Westbrook E.W., Stoots S., Spencer R.F. (1992). Changes in the acoustic nerve after hair cell regeneration. Exp. Neurol..

[bib283] Sanchez-Calderon H., Milo M., Leon Y., Varela-Nieto I. (2007). A network of growth and transcription factors controls neuronal differentation and survival in the developing ear. Int. J. Dev. Biol..

[bib284] Sandell L., Butler Tjaden N., Barlow A., Trainor P. (2014). Cochleovestibular nerve development is integrated with migratory neural crest cells. Dev. Biol..

[bib285] Sapède D., Dyballa S., Pujades C. (2012). Cell lineage analysis reveals three different progenitor pools for neurosensory elements in the otic vesicle. J. Neurosci..

[bib286] Sapède D., Pujades C. (2010). Hedgehog signaling governs the development of otic sensory epithelium and its associated innervation in zebrafish. J. Neurosci.

[bib287] Sato K., Suzuki N. (2001). Whole-cell response characteristics of ciliated and microvillous olfactory receptor neurons to amino acids, pheromone candidates and urine in rainbow trout. Chem. Senses.

[bib288] Sato Y., Miyasaka N., Yoshihara Y. (2005). Mutually exclusive glomerular innervation by two distinct types of olfactory sensory neurons revealed in transgenic zebrafish. J. Neurosci..

[bib289] Satoh T., Fekete D. (2005). Clonal analysis of the relationships between mechanosensory cells and the neurons that innervate them in the chicken ear. Development.

[bib290] Saxena A., Peng B.N., Bronner M. (2013). Sox10-dependent neural crest origin of olfactory microvillous neurons in zebrafish. eLife.

[bib291] Schlosser G. (2006). Induction and specification of cranial placodes. Dev. Biol..

[bib292] Schlosser G. (2010). Making senses: development of vertebrate cranial placodes. Int. Rev. Cell Mol. Biol..

[bib293] Schuck J.B., Smith M.E. (2009). Cell proliferation follows acoustically-induced hair cell bundle loss in the zebrafish saccule. Hear. Res..

[bib294] Schuknecht H.F., Gacek M.R. (1993). Cochlear pathology in presbycusis. Ann. Otol. Rhinol. Laryngol..

[bib295] Schwander M., Kachar B., Müller U. (2010). Review series: the cell biology of hearing. J. Cell Biol..

[bib296] Schwanzel-Fukuda M., Pfaff D. (1989). Origin of luteinizing hormone-releasing hormone neurons. Nature.

[bib297] Serizawa S., Miyamichi K., Nakatani H., Suzuki M., Saito M., Yoshihara Y., Sakano H. (2003). Negative feedback regulation ensures the one receptor-one olfactory neuron rule in mouse. Science.

[bib298] Shailam R., Lanford P.J., Dolinsky C.M., Norton C.R., Gridley T., Kelley M.W. (1999). Expression of proneural and neurogenic genes in the embryonic mammalian vestibular system. J. Neurocytol..

[bib299] Shetty R., Bose S., Nickell M., McIntyre J., Hardin D., Harris A., McClintock T. (2005). Transcriptional changes during neuronal death and replacement in the olfactory epithelium. Mol. Cell. Neurosci..

[bib300] Shi F., Corrales C.E., Liberman M.C., Edge A.S. (2007). BMP4 induction of sensory neurons from human embryonic stem cells and reinnervation of sensory epithelium. Eur. J. Neurosci..

[bib301] Shi F., Edge A.S. (2013). Prospects for replacement of auditory neurons by stem cells. Hear. Res..

[bib302] Shi F., Hu L., Edge A.S. (2013). Generation of hair cells in neonatal mice by beta-catenin overexpression in Lgr5-positive cochlear progenitors. Proc. Natl. Acad. Sci. USA.

[bib303] Shi F., Kempfle J.S., Edge A.S. (2012). Wnt-responsive Lgr5-expressing stem cells are hair cell progenitors in the cochlea. J. Neurosci..

[bib304] Shou J., Murray R., Rim P., Calof A. (2000). Opposing effects of bone morphogenetic proteins on neuron production and survival in the olfactory receptor neuron lineage. Development.

[bib305] Shou J., Rim P., Calof A. (1999). BMPs inhibit neurogenesis by a mechanism involving degradation of a transcription factor. Nat. Neurosci..

[bib306] Shou J., Zheng J.L., Gao W.Q. (2003). Robust generation of new hair cells in the mature mammalian inner ear by adenoviral expression of Hath1. Mol. Cell. Neurosci..

[bib307] Shykind B.M., Rohani S.C., O’Donnell S., Nemes A., Mendelsohn M., Sun Y., Axel R., Barnea G. (2004). Gene switching and the stability of odorant receptor gene choice. Cell.

[bib308] Sienknecht U., Fekete D. (2008). Comprehensive Wnt-related gene expression during cochlear duct development in chicken. J. Comp. Neurol..

[bib309] Sjödal M., Edlund T., Gunhaga L. (2007). Time of exposure to BMP signals plays a key role in the specification of the olfactory and lens placodes ex vivo. Dev. Cell.

[bib310] Slattery E.L., Speck J.D., Warchol M.E. (2009). Epigenetic influences on sensory regeneration: histone deacetylases regulate supporting cell proliferation in the avian utricle. J. Assoc. Res. Otolaryngol..

[bib311] Smith M.E., Coffin A.B., Miller D.L., Popper A.N. (2006). Anatomical and functional recovery of the goldfish (*Carassius auratus*) ear following noise exposure. J. Exp. Biol..

[bib312] Sobkowicz H.M., August B.K., Slapnick S.M. (1997). Cellular interactions as a response to injury in the organ of Corti in culture. Int. J. Dev. Neurosci..

[bib313] Staecker H., Praetorius M., Baker K., Brough D.E. (2007). Vestibular hair cell regeneration and restoration of balance function induced by math1 gene transfer. Otol. Neurotol..

[bib314] Stevens C., Davies A., Battista S., Lewis J., Fekete D. (2003). Forced activation of Wnt signaling alters morphogenesis and sensory organ identity in the chicken inner ear. Dev. Biol..

[bib315] Steyger P.S., Burton M., Hawkins J.R., Schuff N.R., Baird R.A. (1997). Calbindin and parvalbumin are early markers of non-mitotically regenerating hair cells in the bullfrog vestibular otolith organs. Int. J. Dev. Neurosci..

[bib316] Stone J.S., Choi Y.S., Woolley S.M., Yamashita H., Rubel E.W. (1999). Progenitor cell cycling during hair cell regeneration in the vestibular and auditory epithelia of the chick. J. Neurocytol..

[bib317] Stone J.S., Cotanche D.A. (1994). Identification of the timing of S phase and the patterns of cell proliferation during hair cell regeneration in the chick cochlea. J. Comp. Neurol..

[bib318] Stone J.S., Cotanche D.A. (2007). Hair cell regeneration in the avian auditory epithelium. Int. J. Dev. Biol..

[bib319] Strominger R.N., Bohne B.A., Harding G.W. (1995). Regenerated nerve fibers in the noise-damaged chinchilla cochlea are not efferent. Hear. Res..

[bib320] Sullivan S.L., Ressler K.J., Buck L.B. (1995). Spatial patterning and information coding in the olfactory system. Curr. Opin. Genet. Dev.

[bib321] Sun H., Lin C., Smith M. (2011). Growth hormone promotes hair cell regeneration in the zebrafish (*Danio rerio*) inner ear following acoustic trauma. PLoS One.

[bib322] Sun S.-K., Dee C.T., Tripathi V.B., Rengifo A., Hirst C.S., Scotting P.J. (2007). Epibranchial and otic placodes are induced by a common Fgf signal, but their subsequent development is independent. Dev. Biol..

[bib323] Suzuki J., Yoshizaki K., Kobayashi T., Osumi N. (2013). Neural crest-derived horizontal basal cells as tissue stem cells in the adult olfactory epithelium. Neurosci. Res..

[bib324] Sweet E.M., Vemaraju S., Riley B.B. (2011). Sox2 and Fgf interact with Atoh1 to promote sensory competence throughout the zebrafish inner ear. Dev. Biol..

[bib325] Tateya T., Imayoshi I., Tateya I., Hamaguchi K., Torii H., Ito J., Kageyama R. (2013). Hedgehog signaling regulates prosensory cell properties during the basal-to-apical wave of hair cell differentiation in the mammalian cochlea. Development.

[bib326] Taylor R., Forge A. (2005). Hair cell regeneration in sensory epithelia from the inner ear of a urodele amphibian. J. Comp. Neurol..

[bib327] Terayama Y., Kaneko K., Tanaka K., Kawamoto K. (1979). Ultrastructural changes of the nerve elements following disruption of the organ of Corti. II. Nerve elements outside the organ of Corti. Acta Oto-Laryngol..

[bib328] Terayama Y., Kaneko Y., Kawamoto K., Sakai N. (1977). Ultrastructural changes of the nerve elements following disruption of the organ of Corti. I. Nerve elements in the organ of Corti. Acta Oto-Laryngol..

[bib329] Tessarollo L., Coppola V., Fritzsch B. (2004). NT-3 replacement with brain-derived neurotrophic factor redirects vestibular nerve fibers to the cochlea. J. Neurosci..

[bib330] Theriault F., Nuthall H., Dong Z., Lo R., Barnabe-Heider F., Miller F., Stifani S. (2005). Role for Runx1 in the proliferation and neuronal differentiation of selected progenitor cells in the mammalian nervous system. J. Neurosci..

[bib331] Thisse, C., Thisse, B., 2005. High Throughput Expression Analysis of ZF-Models Consortium Clones. ZFIN Direct Data Submission, 〈http://zfin.org〉.

[bib332] Tong M., Brugeaud A., Edge A.S. (2013). Regenerated synapses between postnatal hair cells and auditory neurons. J. Assoc. Res. Otolaryngol..

[bib333] Treloar H., Miller A., Ray A., Greer C., Menini A. (2010). Development of the olfactory system. The Neurobiology of Olfaction.

[bib334] Tucker E., Lehtinen M., Maynard T., Zirlinger M., Dulac C., Rawson N., Pevny L., Lamantia A. (2010). Proliferative and transcriptional identity of distinct classes of neural precursors in the mammalian olfactory epithelium. Development.

[bib335] Valverde F., Heredia M., Santacana M. (1993). Characterization of neuronal cell varieties migrating from the olfactory epithelium during prenatal development in the rat. Immunocytochemical study using antibodies against olfactory marker protein (OMP) and luteinizing hormone–releasing hormone (LH–RH). Brain Res. Dev. Brain Res..

[bib336] Vassar R., Ngai J., Axel R. (1993). Spatial segregation of odorant receptor expression in the mammalian olfactory epithelium. Cell.

[bib337] Vemaraju S., Kantarci H., Padanad M.S., Riley B.B. (2012). A spatial and temporal gradient of Fgf differentially regulates distinct stages of neural development in the zebrafish inner ear. PLoS Genet..

[bib338] Wada N., Javidan Y., Nelson S., Carney T., Kelsh R., Schilling T. (2005). Hedgehog signaling is required for cranial neural crest morphogenesis and chondrogenesis at the midline in the zebrafish skull. Development.

[bib339] Walenkamp M., Wit J. (2007). Genetic disorders in the GH IGF-I axis in mouse and man. Eur. J. Endocrinol..

[bib340] Wang Q., Green S.H. (2011). Functional role of neurotrophin-3 in synapse regeneration by spiral ganglion neurons on inner hair cells after excitotoxic trauma in vitro. J. Neurosci..

[bib341] Warchol M.E., Corwin J.T. (1996). Regenerative proliferation in organ cultures of the avian cochlea: identification of the initial progenitors and determination of the latency of the proliferative response. J. Neurosci..

[bib342] Warchol M.E., Lambert P.R., Goldstein B.J., Forge A., Corwin J.T. (1993). Regenerative proliferation in inner ear sensory epithelia from adult guinea pigs and humans. Science.

[bib343] Webster D.B., Webster M. (1982). Multipolar spiral ganglion neurons following organ of Corti loss. Brain Res..

[bib344] Webster M., Webster D. (1981). Spiral ganglion neuron loss following organ of Corti loss: a quantitative study. Brain Res..

[bib345] Weisleder P., Rubel E.W. (1992). Hair cell regeneration in the avian vestibular epithelium. Exp. Neurol..

[bib346] Weisleder P., Rubel E.W. (1993). Hair cell regeneration after streptomycin toxicity in the avian vestibular epithelium. J. Comp. Neurol..

[bib347] Wersäll J. (1956). Studies on the structure and innervation of the sensory epithelium of the cristae ampullares in the guinea pig; a light and electron microscopic investigation. Acta Oto-Laryngol..

[bib348] Whitfield T.T., Riley B.B., Chiang M.-Y., Phillips B. (2002). Development of the zebrafish inner ear. Dev. Dyn..

[bib349] Whitlock K. (2004). Development of the nervus terminalis: origin and migration. Microsc. Res. Tech..

[bib350] Whitlon D., Gabel C., Zhang X. (1996). Cochlear inner hair cells exist transiently in the fetal Bronx Waltzer (bv/bv) mouse. J. Comp. Neurol..

[bib351] Woods C., Montcouquiol M., Kelley M.W. (2004). Math1 regulates development of the sensory epithelium in the mammalian cochlea. Nat. Neurosci..

[bib352] Wray S., Grant P., Gainer H. (1989). Evidence that cells expressing luteinizing hormone–releasing hormone mRNA in the mouse are derived from progenitor cells in the olfactory placode. Proc. Natl. Acad. Sci. USA.

[bib353] Wray S., Nieburgs A., Elkabes S. (1989). Spatiotemporal cell expression of luteinizing hormone-releasing hormone in the prenatal mouse: evidence for an embryonic origin in the olfactory placode. Brain Res. Dev. Brain Res..

[bib354] Wu D.K., Oh S.-H. (1996). Sensory organ generation in the chick inner ear. J. Neurosci..

[bib355] Wu H., Ivkovic S., Murray R., Jaramillo S., Lyons K., Johnson J., Calof A. (2003). Autoregulation of neurogenesis by GDF11. Neuron.

[bib356] Xu J., Huang D., Hou Z., Guo W., Sun J., Zhao L., Yu N., Young W., He D., Yang S. (2012). Type I hair cell regeneration induced by Math1 gene transfer following neomycin ototoxicity in rat vestibular sensory epithelium. Acta Oto-Laryngol..

[bib357] Yamamoto N., Tanigaki K., Tsuji M., Yabe D., Ito J., Honjo T. (2006). Inhibition of Notch/RBP-J signaling induces hair cell formation in neonate mouse cochleas. J. Mol. Med. (Berl).

[bib358] Yamasoba T., Kondo K. (2006). Supporting cell proliferation after hair cell injury in mature guinea pig cochlea in vivo. Cell Tissue Res..

[bib359] Yang S., Chen W., Guo W., Jia S., Sun J., Liu H., Young W., He D. (2012). Regeneration of stereocilia of hair cells by forced Atoh1 expression in the adult mammalian cochlea. PLoS One.

[bib360] Yuan Y., Shi F., Yin Y., Tong M., Lang H., Polley D.B., Liberman M.C., Edge A.S. (2014). Ouabain-induced cochlear nerve degeneration: synaptic loss and plasticity in a mouse model of auditory neuropathy. J. Assoc. Res. Otolaryngol..

[bib361] Zakir M., Dickman J.D. (2006). Regeneration of vestibular otolith afferents after ototoxic damage. J. Neurosci..

[bib362] Zhang X., Firestein S. (2009). Genomics of olfactory receptors. Res. Prob. Cell Diff..

[bib363] Zhang X., Rogers M., Tian H., Zhang X., Zou D.J., Liu J., Ma M., Shepherd G.M., Firestein S.J. (2004). High-throughput microarray detection of olfactory receptor gene expression in the mouse. Proc. Natl. Acad. Sci. USA.

[bib364] Zheng J.L., Gao W.-Q. (2000). Overexpression of Math1 induces robust production of extra hair cells in postnatal rat inner ears. Nat. Neurosci..

